# Chemical Compensation Challenges in Processing Antiferroelectric PbZrO_3_ Thin Films

**DOI:** 10.1021/acsomega.5c04818

**Published:** 2025-07-05

**Authors:** Milan H. Haddad, Vasily Lebedev, Kristina Holsgrove, Sergio Rivera-Cruz, Sarah Stock, Nikhilesh Maity, Sergey Lisenkov, Inna Ponomareva, Amit Kumar, Lewys Jones, Nazanin Bassiri-Gharb

**Affiliations:** † School of Materials Science and Engineering, 1372 Georgia Institute of Technology, Atlanta, Georgia 30332, United States; ‡ School of Physics, Trinity College Dublin, College Green, Dublin 2, Ireland; § Advanced Microscopy Laboratory, Centre for Research on Adaptive Nanostructures & Nanodevices (CRANN), Trinity College Dublin, Dublin 2, Ireland; ∥ Centre for Quantum Materials and Technologies, School of Mathematics and Physics, 1596 Queen’s University Belfast, Main Physics Building, University Road, Belfast, Northern Ireland BT7 1NN, U.K.; ⊥ Electrical and Computer Engineering Department, University of Puerto Rico-Mayaguez, Mayaguez 00680, Puerto Rico; # Department of Physics, 7831 University of South Florida, Tampa, Florida 33620, United States; ¶ G.W. Woodruff School of Mechanical Engineering, 1372 Georgia Institute of Technology, Atlanta, Georgia 30332-0405, United States

## Abstract

The electric field-induced antipolar-to-polar phase transition in antiferroelectric materials is accompanied by large changes in the structural and functional response, making them attractive for many applications ranging from pulsed energy capacitors to high-strain-high (blocking-)­force actuators, solid-state heating and cooling systems, and optoelectronic devices. PbZrO_3_, as an end member of the PZT, (*x*)­PbZr_1–*x*_Ti_*x*_O_3_, solid solution, has been the subject of many studies. However, processing of perovskite PbZrO_3_ is extremely challenging due to phase instabilities induced by Pb loss at the temperatures necessary for perovskite crystallization. Here, we discuss the challenges associated with Pb loss, as well as its compensation through bulk Pb overstoichiometry and interfacial PbO additions, in chemical solution processed, highly oriented PbZrO_3_ thin films. For both 042_o_- and 001_o_-oriented (*o*-orthorhombic distortion) PbZrO_3_ thin films, the crystallization interfaces are the most important contributors to Pb loss and off-stoichiometric outcomes, and no single approach was sufficient to address these challenges. Pb-rich and Pb-deficient nonperovskite phases including ZrO_*x*_ were only observable through microscopic characterization, and X-ray diffraction alone could not rule out the presence of such secondary phases. “Ideal” macroscopic antiferroelectric polarization-electric field double hysteresis curves were observed despite (at times) the large presence of secondary phases. However, reduced saturation polarization and increased phase transition electric fields were observed concomitantly with the presence of secondary phase(s) and tentatively assigned to the voltage drop across the ZrO_*x*_ nanocrystals. Based on these results, it is imperative that the presence of secondary phases always be addressed (beyond X-ray diffraction spectra) in order to correctly evaluate the properties of antiferroelectric films.

## Graphical abstract

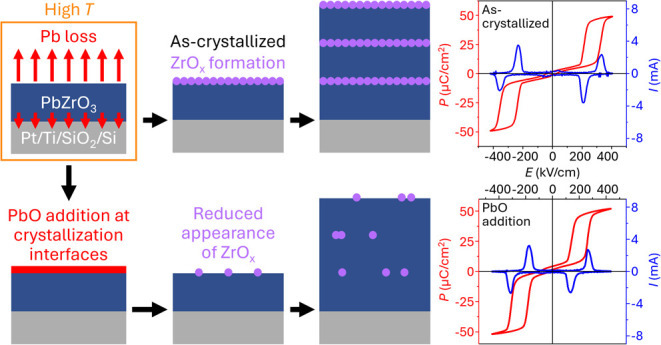

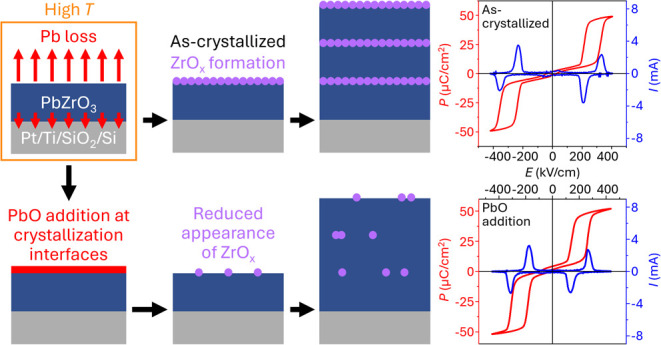

## Introduction

Antiferroelectric materials undergo an antipolar-to-polar transition under the application of sufficiently high electric fields. The changes in functional properties which accompany this phase transition enable a wide range of applications including pulsed energy capacitors, high strain-high (blocking-)­force actuators, solid-state heating and cooling systems, and optoelectronic modulators.^1^ PbZrO_3_, the first discovered and widely considered prototypical antiferroelectric material, is an end member of the technologically ubiquitous PZT, PbZr_1–*x*_Ti_*x*_O_3_, solid solution. While processing of PZT has been studied extensively,^2–6^ PbZrO_3_ remains extremely challenging to process due to the instability of the perovskite phase.^7^ Furthermore, this material often suffers from dielectric breakdown fields lower than the critical electric fields required for antipolar-to-polar phase transition (*E*_f_).^7^ A- and B-site codoping has proved an effective strategy for circumventing this challenge by decreasing the phase transition electric fields for bulk ceramics.^8^ On the other hand, thin films can withstand higher electric fields compared to bulk samples because of their relatively higher density and more uniform microstructure.^9^ While A and B site codoping is still frequently used for tailoring dielectric properties of PbZrO_3_ thin films,^10^ consistent and homogeneous chemical doping is more challenging to achieve for the smaller grains of polycrystalline thin films compared to bulk samples.^11^

A major challenge in processing “high quality” perovskite PbZrO_3_ thin films, and in general many Pb-based oxide perovskites, is Pb loss at the high temperatures required for crystallization, often at ∼700 °C. Specifically, the effects of Pb loss on the microstructure, phase development, and dielectric properties of PZT have been extensively studied since the 1990s.^4–6,12^ As the mechanisms of Pb loss have not been reported specifically with respect to PbZrO_3_, we discuss below those reported for other Pb-based perovskite ferroelectric films and, in particular, those processed by chemical solution processing. During pyrolysis of the amorphous film, decomposition of the organic content results in a locally oxygen-poor environment, which reduces the organometallic Pb to metallic Pb^0^.^11^ For PZT thin films pyrolyzed in air, oxidation of metallic Pb^0^ resulting in the formation of PbO has been reported to occur at ∼400 °C.^13^ Volatilization of PbO occurs at higher temperatures, for example, starting from 450 °C for the Pb_1–*x*_La_*x*_TiO_3_ system, and the quantity of Pb lost increases with increasing annealing temperature.^14^ For PbZrO_3_, PbO volatilization is expected to result in the formation of Pb and O vacancies according to the following defect reactions

1 $${Pb}_{Pb}^{x}+{Zr}_{Zr}^{x}+3O_{O}^{x}⇌{PbO}_{\left(g\right)}+{Zr}_{Zr}^{x}+2O_{O}^{x}+V_{Pb}^{\prime \prime }+V_{O}^{\cdot \cdot }+2h^{\cdot }$$

PbO volatilization has been reported to occur up to 5 nm beneath the surface of PZT films.^15^ For processing PZT-based thin films, various Pb compensation approaches have been reported, including Pb overstoichiometry for bulk Pb losses^16^ and Pb compensation directly at the interfaces.^17,18^ Bulk ceramic Pb-based perovskites are also frequently processed by sintering in the presence of excess PbO powder to increase the PbO vapor pressure in proximity of the powdered oxides.^19^ However, thin films experience greater Pb loss than bulk ceramics due to their higher surface-to-volume ratio and, consequentially, require much higher Pb compensation supplied directly to the film. Furthermore, the presence of powders in the crystallization chamber can result in powder transfer to the film surface, resulting in discontinuities and/or dielectric shorts and overall lower film quality. Therefore, the use of a PbO supply through the use of powder beds is excluded from this study.

PbZrO_3_ is reported to experience overall greater Pb loss compared to PZT with higher Ti content (e.g., at the morphotropic phase boundary, MPB, with Zr/Ti of ∼48:52),^20^ but the associated challenges have not been similarly addressed and remain less understood. Considering that the PbO–ZrO_2_ binary system can tolerate <2 at. % Pb deficiency before fluorite phased ZrO_2_ is stabilized,^21^ Pb-deficient phases should be expected to routinely form in PbZrO_3_ films processed with insufficient Pb. This work explores multiple Pb compensation approaches in chemically solution processed PbZrO_3_ thin films. We specifically consider “bulk” and interfacial Pb additions and discuss the effects of Pb loss and its compensation on the microstructure, crystallographic orientation, chemical compositional, and polarization response. Chemical solution deposition was chosen as it offers a low-cost and easily scalable thin film processing technique that enables greater stoichiometric control and chemical homogeneity over a larger area compared to many other thin film deposition techniques.^11^ Furthermore, we used platinized Si wafers, compatible with and routinely used in many microelectromechanical systems based on ferroic perovskite thin films.

## Results and Discussion

A 2-methoxyethanol-based chemical solution route, after Yao et al.,^22^ was leveraged to process PbZrO_3_ films on Pt/TiO_*x*_/SiO_2_/Si substrates. Iterative spin-coating of the in-house processed PbZrO_3_ precursor solution onto the substrate, followed by drying and pyrolysis, was performed, as indicated in Figure 1a. Crystallization was carried out in a rapid thermal annealer (RTA) after every 2 PbZrO_3_ layer depositions (total 3 crystallizations for each sample) to create ∼300 nm thick PbZrO_3_ films. 40 mol % Pb overstoichiometry (which will be referred to henceforth as “% Pb excess”) was used in the precursor solution to compensate for Pb loss during heat treatment steps. The resulting films’ X-ray diffraction (XRD) spectra (Figure 2a) show only peaks assigned to the perovskite phase for lead zirconate (PDF 01-089-1296) or the substrate.^23^ Furthermore, the films are highly 042_o_-oriented (corresponding to pseudocubic 111, 111_pc_), consistent with prior reports of growth of highly 111-oriented MPB PZT films^24^ and 042_o_-oriented PbZrO_3_ deposited on platinized Si substrates.^25–29^

**Figure 1:**
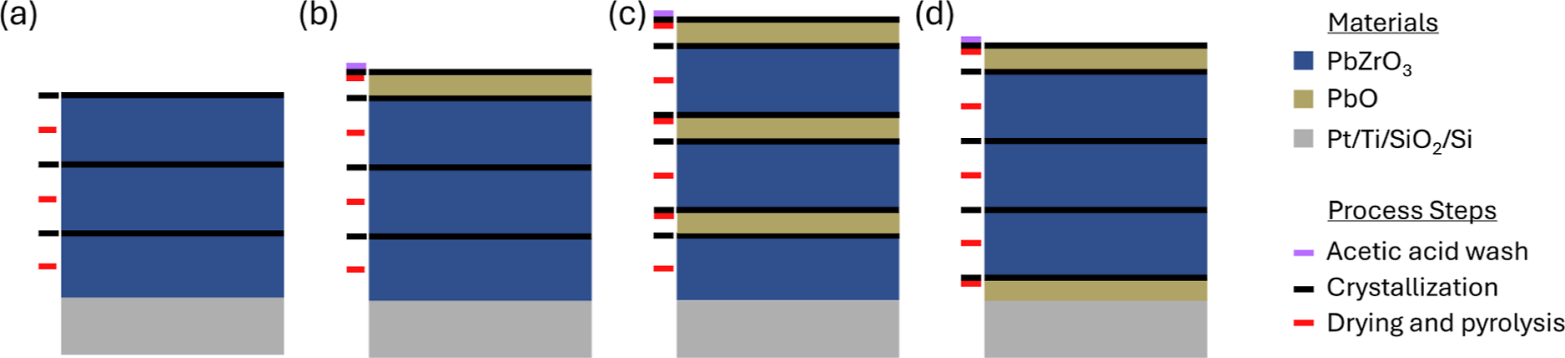
Schematic representation of chemical solution processing steps for deposition of PbZrO_3_ thin films and addition of Pb to compensate for its loss at crystallization through (a) simple Pb addition through precursor solution Pb overstoichiometry; (b) Pb overstoichiometry in the precursor solution and addition of a PbO capping layer; (c) precursor solution Pb overstoichiometry and PbO film deposition after each PbZrO_3_ crystallization; and (d) precursor solution Pb overstoichiometry and PbO used both as a seed layer before the first PbZrO_3_ layer deposition and final capping layer. Black lines indicate crystallization interfaces. Drying and pyrolysis, crystallization, and acetic acid wash steps are shown to the left of each stack at the step they were performed.

**Figure 2:**
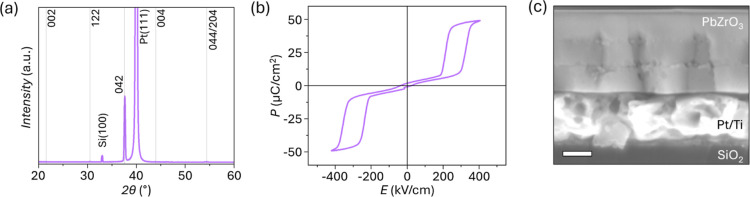
(a) X-ray diffraction pattern, (b) polarization–electric field, *P–E*, response, and (c) cross-sectional scanning electron microscopy (SEM) of PbZrO_3_ processed with 40% Pb excess. The gray lines in (a) correspond to the peak locations of the reference bulk ceramic PbZrO_3_ with peaks indexed according to the orthorhombic perovskite structure taken from PDF 01-089-1296. Scale bar in (c) is 100 nm.

Double-hysteresis loops, characteristic of antiferroelectric materials, are observed in the macroscopic polarization-electric field (*P–E*) measurements performed by using metal–oxide–metal capacitors (Figure 2b). The hysteresis curves are well saturated, with relatively “sharp” saturation polarization at maximum applied fields and minimal opening at fields below the polar-to-antipolar transition electric field, *E*_a_. The switching current–electric field (*I*–*E*) response shows four peaks corresponding to the antipolar-to-polar and polar-to-antipolar phase transitions, which occur at 350 ± 7 kV/cm and 210 ± 4 kV/cm, respectively (Figure S1a). While this functional response is consistent with the absence of nonperovskite peaks in the XRD pattern, scanning electron microscopy (SEM) of the film cross-section revealed nanocrystals with bright contrast, distinctly differing from the bulk of the PbZrO_3_ grains. The nanocrystals appear regularly at specific film depths, corresponding to the buried crystallization interfaces within the film cross-section (Figure 2c) and similarly decorate the film surface (Figure 3a). To discover additional information about these possible secondary phases, grazing incidence XRD (GIXRD) measurements were performed on the as-crystallized PbZrO_3_ films. Only one peak is observed in the GIXRD pattern at 2θ = 57.4°, which did not match perovskite PbZrO_3_ (Figure S2).

**Figure 3:**
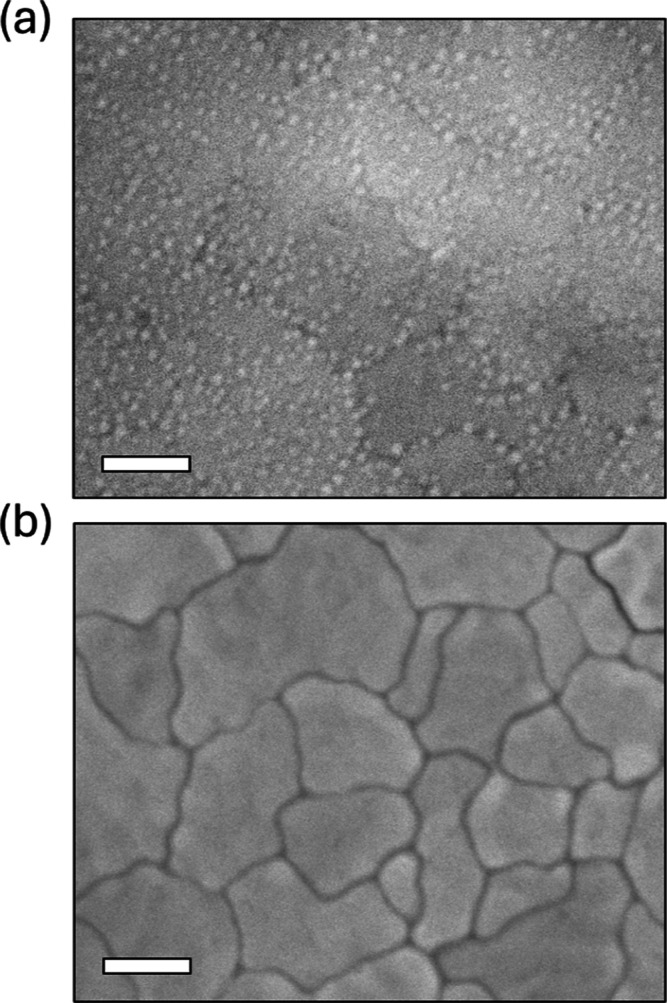
SEM surface images of PbZrO_3_ (a) as-crystallized and (b) after PbO capping. Scale bars are 100 nm.

While it is not possible to identify a (potential secondary) crystallographic phase with a single peak in the diffraction spectrum, the formation of secondary phases due to excessive Pb loss is well documented for other Pb-based perovskites and, particularly, Pb-based ferroelectrics including PZT,^6^ PbTiO_3_,^30^ and Pb­(Mn_1/3_Nb_2/3_)­O_3_.^31^ PbO volatilization in PZT and PZT-based thin films has been reported to result in the creation of pyrochlore (A_2_B_2_O_7_) or disordered fluorite phases (M_2_O_4–*x*_ structure, where M can be A- or B-site atoms) with fewer cation to anion ratios compared to the perovskite phase.^12^ As PbO is lost primarily at the surface (i.e., interface with air), Pb-deficient phases often form at the film surface or at grain boundaries.^16,32,33^ Given the nonpolar nature of pyrochlore and fluorite phases, their presence can affect the overall ferroelectric performance of the film.^6,34^ The amount of such secondary phases can be reduced by compensating for Pb to the film surface specifically. This strategy is achieved by deposition of a PbO solution on the perovskite film followed by a heat treatment (henceforth referred to as a “PbO capping layer”), either before crystallization of the perovskite phase^3,17,18,35^ or afterward.^36,37^ In the former case, Pb compensation reduces or prevents the formation of Pb-deficient phases. In the latter case, the final heat treatment transforms the metastable fluorite phase back into the perovskite. Overall, PbO capping has been reported to improve the phase purity and the ferroelectric properties of PZT-based films.^17^

To evaluate the effects of PbO capping on PbZrO_3_, a single layer of 0.08 M PbO precursor solution was deposited on a sample cut from the as-crystallized PbZrO_3_ film (to improve direct comparisons of the properties) and dried, pyrolyzed, and crystallized (Figure 1b). To remove eventual residual PbO leftover on the surface, the sample surface was washed with acetic acid postcrystallization. The surface of the thus-processed film shows relatively clean grains in SEM (Figure 3b) and is mostly devoid of nanocrystals. Consistent with the outcomes of this approach in PZT films, the observed changes in the surface morphology support the hypothesis of a Pb deficiency at the origin of the nanocrystals. The XRD pattern does not indicate any major variations for the PbZrO_3_ peaks (Figure 4a). The *P*–*E* response of the capped film (Figure 4b) shows a decrease in the antipolar-to-polar transition electric field, *E*_f_, and *E*_a_ by approximately 8% and 11% from 350 ± 7 to 320 ± 5 kV/cm and from 210 ± 4 to 180 ± 2 kV/cm, respectively, compared to the simple lead zirconate film deposition. On the other hand, the saturation polarization increases by approximately 11%—from 45 ± 1 μC/cm^2^ to 50 ± 3 μC/cm^2^—compared to the as-crystallized PbZrO_3_. The *I*–*E* response of the capped film shows similar shifts in the peaks corresponding to *E*_f_ and *E*_a_ in the presence or absence of the PbO capping layer (Figure S1a). Despite the effectiveness of the PbO cap in removing surface nanocrystals, buried nanocrystals are still observed in the cross-sectional SEM images (Figure 4c).

**Figure 4:**
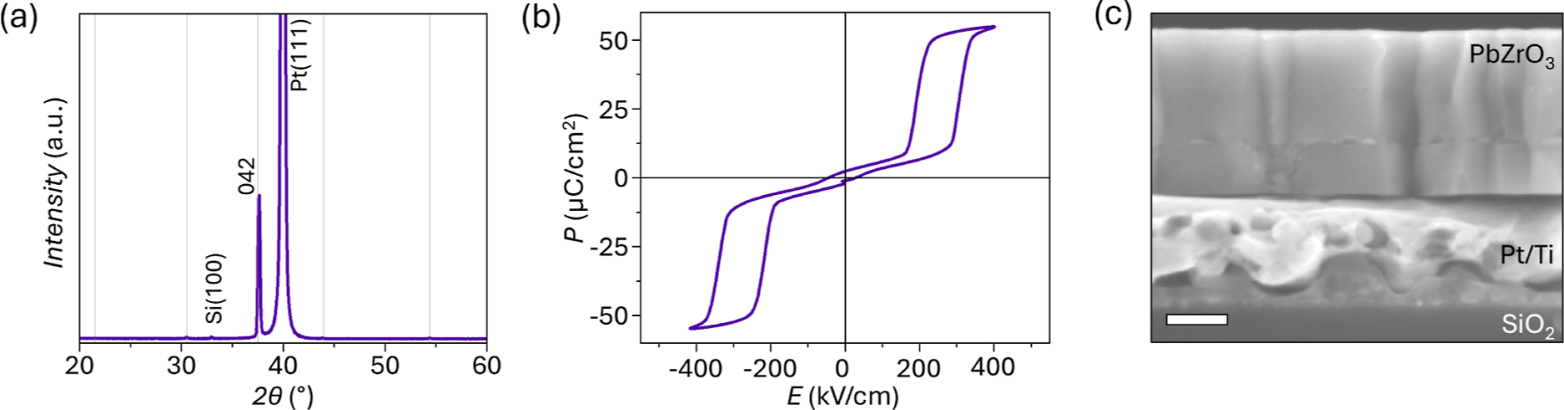
(a) XRD pattern, (b) polarization-electric field response, and (c) cross-sectional SEM image of PbZrO_3_ processed with 40% Pb excess and a 0.08 M PbO cap, crystallized separately and washed off after crystallization with acetic acid. Scale bar in (c) is 100 nm.

The persistence of nanocrystals within the film suggests that the Pb excess in the precursor solution, even with an additional Pb supply to cap the films, is insufficient to fully compensate losses at the previous crystallization interfaces. Hence, “intermediate” PbO layers at each crystallization interface were used next: after each PbZrO_3_ crystallization, a 0.08 M PbO layer was deposited, dried, pyrolyzed, and crystallized at 700 °C for 70 s before deposition of the next PbZrO_3_ layer (Figure 1c). Inclusion of two PbO layers within the films was offset by reduction of the bulk Pb overstoichiometry (provided by the precursor solution) from 40 to 35%. Using PbO compensation after each PbZrO_3_ crystallization did not substantially affect the *P*–*E* curves of the films (Figure 5b); however, it reduced the appearance of buried nanocrystals in the cross-sectional SEM, compared to those observed in PbZrO_3_ films processed without intermediate PbO layers (Figure 5c). This change supports the hypothesis that the buried nanocrystals, like those on the surface, are also Pb-deficient. While PbO capping did not result in major variations of the XRD pattern, for PbZrO_3_ processed with intermediate PbO layers, the XRD pattern showed additional peaks (at 2θ = 21.5° and 43.9°) corresponding to the 002_o_ and 004_o_ reflections of perovskite PbZrO_3_, respectively (Figure 5a). Consequently, in these films, the {021}_o_ Lotgering factor^38^ (LF_021_) was reduced from ∼100% to ∼85%. Chen and Chen have previously discussed the use of large Pb overstoichiometry in the precursor solution leading to the formation of 001-oriented PbO particles at the interface with Pt starting at approximately 300 °C.^39^ These PbO particles were reported to nucleate {001}_o_-oriented perovskite PZT grains through lattice matching during subsequent crystallization.^5^ Similarly, Gong and co-workers have shown that highly {001}_o_-oriented perovskite PZT thin films can be deposited through the use of PbO “seed” layers.^40,41^

**Figure 5:**
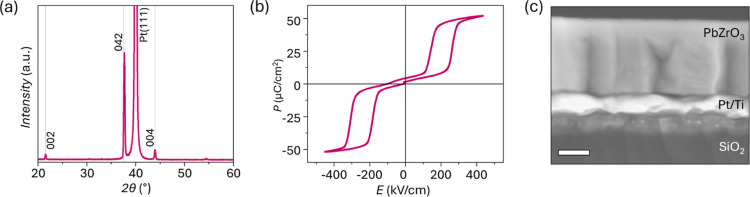
(a) XRD pattern, (b) polarization-electric field response, and (c) cross-sectional SEM image of PbZrO_3_ processed with 35% Pb excess and 0.08 M PbO layers deposited and crystallized after each PbZrO_3_ crystallization step. Scale bar in (c) is 100 nm.

To investigate the correlation between PbO addition and the growth of {001}_o_-oriented PbZrO_3_, a single PbO layer was deposited and crystallized immediately on top of the platinized substrate. Microstructural characterization of this seed layer and the bare Pt bottom electrode is shown in the Supporting Information (Figure S3). PbZrO_3_ films were deposited on the PbO seed layer, using 40% Pb excess, both without and with a 0.08 M PbO cap (Figure 1d). The as-crystallized films showed strong preferential 001_o_ orientation, with LF_001_ of 92% (Figure 6a). After PbO capping, the LF_001_ increased to 97% (Figure 6a). Such high {001}_o_ orientation of PbZrO_3_ with the use of a PbO seed is consistent with previous work by a subset of the authors.^26^

**Figure 6:**
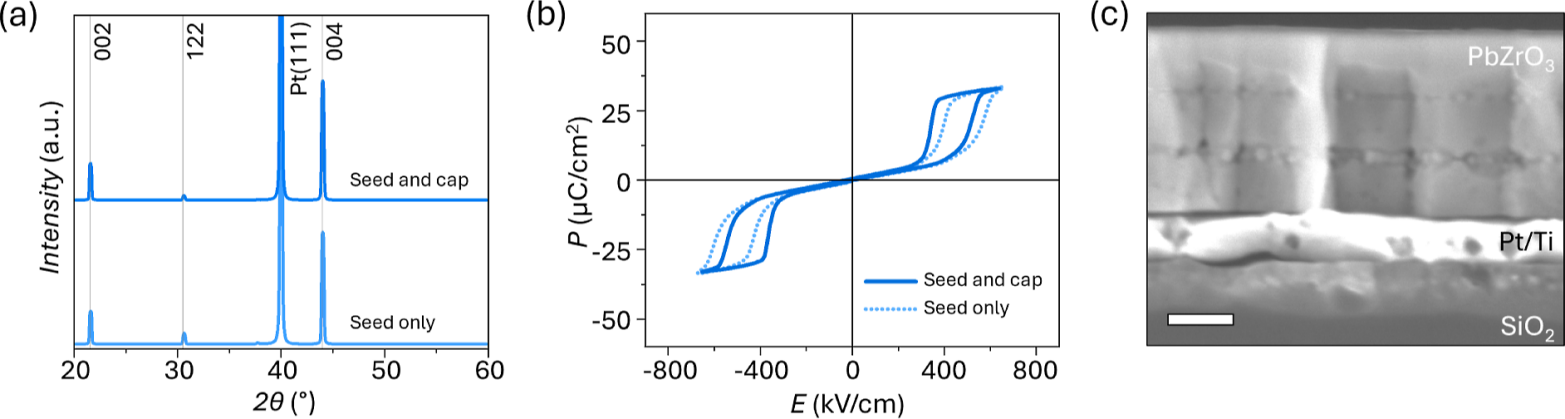
(a) XRD patterns and (b) polarization–electric field responses of PbZrO_3_ films processed with a 0.14 M PbO seed and 40% Pb excess, without additional Pb compensation and with a 0.08 M PbO cap deposited and crystallized after the last PbZrO_3_ crystallization, and (c) cross-sectional SEM image of the film processed with a 0.14 M PbO seed, before deposition of the 0.08 M PbO cap. Scale bar in (c) is 100 nm.

Compared to the films grown directly on the platinized substrates, the *P*–*E* responses of the PbO-seeded PbZrO_3_ (Figure 6b) show an increase of *E*_f_ and *E*_a_—for the as-crystallized films, from 350 ± 7 kV/cm to 570 ± 8 kV/cm and from 210 ± 4 kV/cm to 380 ± 3 kV/cm, respectively, and for the PbO capped films, from 320 ± 5 kV/cm to 520 ± 10 kV/cm and from 180 ± 2 kV/cm to 330 ± 4 kV/cm, respectively. Table S1 summarizes the transition electric fields, saturation polarization, Lotgering factors, and thicknesses of all five PbZrO_3_ films. The changes observed in the phase transition electric fields with changes in the crystallographic orientation are also consistent with the previous reports on anisotropic response of PbZrO_3_ thin films.^26,42–46^ Addition of a PbO cap to the PbO seeded film resulted in sharper antipolar-to-polar and polar-to-antipolar transitions in the *P*–*E* curves. The narrower range of electric fields resulting in phase transition, consistent with the presence of a narrower range of crystallographic orientations, is also accompanied by an effective decrease of *E*_f_ and *E*_a_, as mentioned above, from 570 ± 8 kV/cm to 520 ± 10 kV/cm and from 380 ± 3 kV/cm to 330 ± 4 kV/cm, respectively (Figure 6b). In PbO-seeded PbZrO_3_ films, a small additional pair of antipolar-to-polar phase transition peaks is observed in the *I*–*E* loops at ∼± 400 kV/cm (Figure S1b). These peaks are not observed in the switching current loops of the 042_o_-oriented films (Figure S1a,c), and they are similarly distinct from the higher intensity pair of peaks expected to correspond to the polarization switching of the {001}_o_-oriented grains. Considering the appearance of the perovskite 122_o_ XRD peak (Figure 6a) in the PbO-seeded films, the additional switching current peaks are attributed to the polarization switching of 122_o_- or randomly oriented grains. Despite the higher molarity of PbO seed layer precursor compared to the PbO solution used for the intermediate compensation at the crystallization interface, the films processed with the seed layer showed once again the presence of nanocrystals with bright contrast in SEM cross-sectional analysis (Figure 6c). This observation suggests possible additional Pb loss not only at the surface of the films but possibly also to the substrate.

To verify possible Pb loss into the substrate and at each crystallization interface, the film’s cross-section’s chemical composition was mapped using high-angle annular dark-field scanning transmission electron microscopy (HAADF–STEM) energy dispersive X-ray spectroscopy (EDS). Intriguingly, no clean interfaces are observed between various chemical species at the bottom electrode/substrate: Pt, Ti, Pb, and Si all show signs of relative interdiffusion with Pt pockets appearing within and below the Ti layer (Figure 7a). Furthermore, a ∼30 nm thick Pb-rich layer is observed beneath the Ti layer (Figure 7a) supporting the hypothesis that Pb diffuses through the bottom electrode stack of Pt/Ti further into the substrate and the SiO_2_ layer.^6^

**Figure 7:**
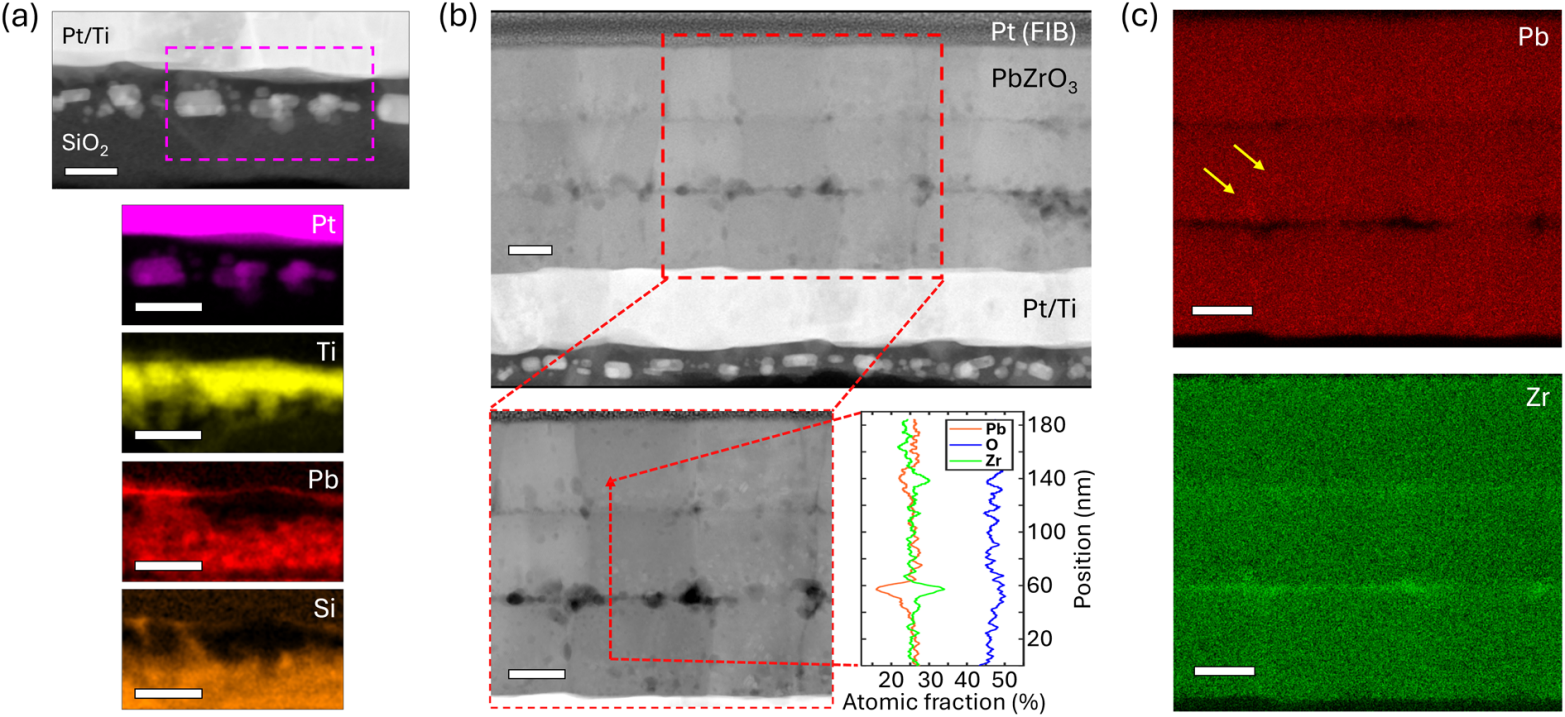
(a) High-angle annular dark-field scanning transmission electron microscopy (HAADF-STEM) and corresponding energy dispersive X-ray spectroscopy (EDS) maps of the cross-sectional interface between the Pt/Ti bottom electrode and the SiO_2_ substrate beneath a PbZrO_3_ film processed with 40% Pb excess and a 0.14 M PbO seed layer, (b) HAADF–STEM image and EDS line profile (marked by a dashed red line), and (c) maps from the film cross-section as in (a) and corresponding to the boxed region in (b). The X-ray emission lines used were Pt L-α, Ti K-α, Pb L-α, Si K-α, Zr K-α, and O K-α. Scale bars are 50 nm across all panels.

Within the film thickness, the EDS line scan (Figure 7b) and area maps (Figure 7c) show a lower atomic concentration of Pb at two specific film thicknesses compared to the bulk of the film. The instances of such Pb deficiency, both in number and configuration (parallel to the substrate and film surface), are consistent with a continuous and stepwise repeated loss of Pb and, therefore, PbO volatilization from the film’s free surface at each crystallization. Hence, despite excess Pb provided through precursor solution off-stoichiometry and additional Pb provided by the seed layer, the films remain Pb-deficient and locally Zr-rich, particularly in proximity of intermediate PbZrO_3_ crystallization interfaces. High-resolution HAADF–STEM analysis shows that this Pb-depletion layer does not disrupt growth of the perovskite PbZrO_3_ film across the crystallization interfaces with a continuous crystal structure (Figure S4). In addition to the Pb depletion layer, Pb-rich “pockets” of material are also observed locally, as indicated by the yellow arrows in Figure 7c. Additional EDS analysis of such inclusions confirms their Pb enrichment (Figure S5). Considering that each crystallized PbZrO_3_ layer is expected to supply Pb to the underlying Pb-depleted crystallization surface, the formation of a Pb-rich phase in close proximity of the Zr-rich phase is unexpected and further suggests that the Zr-rich phase cannot be transformed to perovskite by Pb excess provided only through the precursor.

To gain further insight into the Pb-deficient nanocrystals, detailed high-resolution STEM (HR-STEM) analysis of the microstructural heterogeneities observed in the film’s SEM cross-section in proximity of the crystallization interfaces was performed (Figure 8). We specifically compare a single nanocrystal (Figure 8b) with the bulk PbZrO_3_ used as a reference (Figure 8c). The spatial distribution of atomic columns was analyzed and compared with those of multiple structures of ZrO_*x*_ and PbO_*x*_. The steps of the analysis are described in detail in the Supporting Information (Figures S6–S9). Based on the observed values of interatomic distances and systematic displacements, the orthorhombic Ortho-I^47,48^ phase of ZrO_*x*_ (space group *Pbcm*) is proposed as the best match. The proposed structures and corresponding simulated HAADF–STEM images for ZrO_*x*_ and PbZrO_3_ are shown in Figure 8d–g. The presence of ZrO_*x*_ in the final structure despite the observation of Pb-rich proximal volumes can be explained by the stability of ZrO_2_ in the PbO–ZrO_2_ binary system up to 1538 °C.^49^ It is possible that the ZrO_2_ nanocrystals are initially larger than they appear in the final structure and are partially transformed into perovskite during crystallization of subsequent PbZrO_3_ layers by partial reaction with Pb excess in the “next” deposited layer. In this case, if the Pb excess in the proximity of the ZrO_*x*_ nanocrystals is depleted, the ZrO_2_ inclusions observed by HR-STEM would be leftover ZrO_*x*_, which did not receive sufficient Pb supply to transform into perovskite.

**Figure 8:**
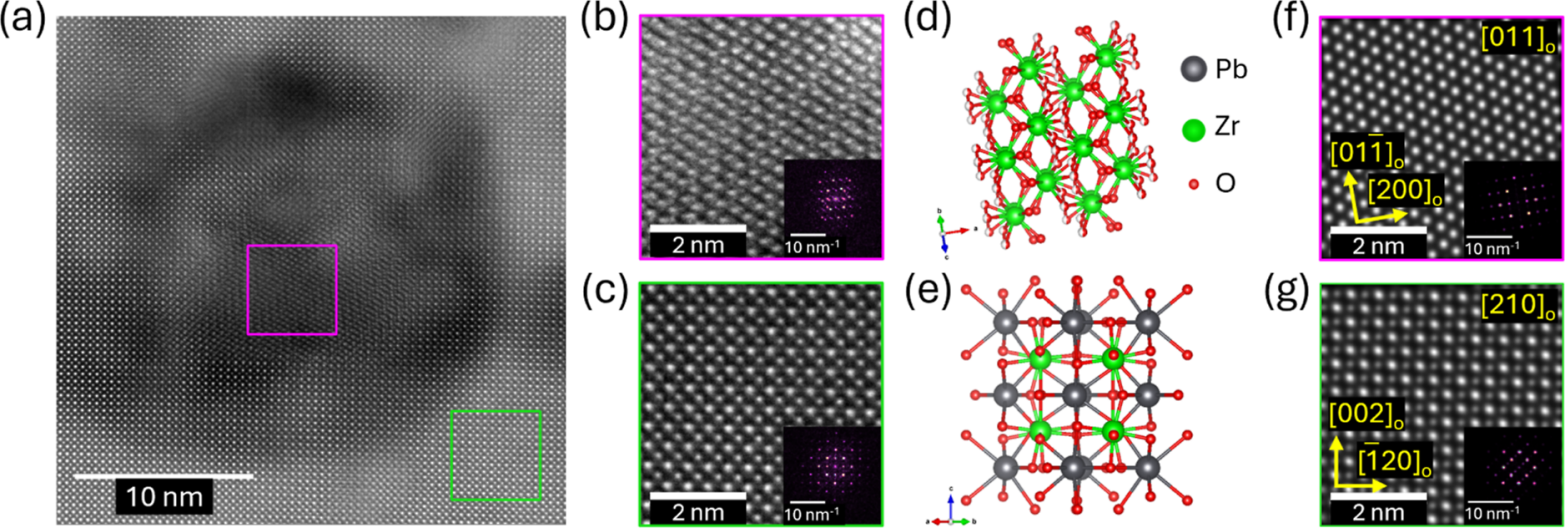
High-resolution STEM (HR-STEM) analysis of an inclusion in PbZrO_3_ processed with 40% Pb excess and a 0.14 M PbO seed: (a) nonrigidly aligned multiframe HAADF–STEM image with drift correction and cropped image segments of (b) the inclusion area and (c) the PbZrO_3_ matrix. Schematics of the suggested orthorhombic (d) ortho-I ZrO_2_ and (e) PbZrO_3_ structures were plotted using VESTA; atom labels for both structures are shown in (d). (f,g) Simulated HAADF–STEM images for the proposed structures were generated using the abTEM software. Scale bar lengths are indicated on each relevant panel.

In multiple literature reports on PbZrO_3_, despite observation of nanocrystals with bright SEM contrast, their presence—and therefore, their composition, crystallographic phase, and potential influence on the functional response—is left unaddressed; often, the PbZrO_3_ films are reported to be pure perovskite based on the absence of secondary-phased XRD peaks.^27,42,50–53^ Most recently, Wen and co-workers^54^ identified ZrO_2_ nanocrystals on the surfaces and in proximity of buried crystallization interfaces of films prepared from (under-stoichiometric or) Pb-deficient PbZrO_3_ precursor solutions. Similar to the present work, all peaks observed in the XRD data were assigned to the perovskite. The consistent absence of ZrO_2_ peaks in XRD patterns of PbZrO_3_ thin films possibly stems from a 2-fold challenge. First, the nanocrystals are often smaller than ∼10 nm, approaching the detection limit in laboratory-based diffraction tools. Second, the highest intensity peak of the Ortho-I ZrO_2_ phase identified here coincides with the peak corresponding to the 122_o_ reflection of perovskite PbZrO_3_ at 2θ = 30.2°.^48^ Considering these limitations in XRD detection of ZrO_2_, diffraction methods will be insufficient for complete evaluation of phase purity in PbZrO_3_ thin films.

We now consider the effects of the presence of ZrO_2_ nanocrystals on the functional properties of the films discussed above. In the first approximation, ZrO_2_ nanocrystals can be considered a single layer of material with different dielectric properties than the bulk of the PbZrO_3_ film. Thus, under the measurement conditions, we can consider two capacitors in series representing zirconia and lead zirconate (Figure S9). The dielectric permittivity of ZrO_2_ is lower compared to that of PbZrO_3_. Therefore, the presence of a thin (relative to the PbZrO_3_ film thickness) ZrO_2_ layer(s) would result in a voltage drop across this layer, effectively decreasing the voltage and, hence, the electric field across PbZrO_3_. Consequentially, the measured *E*_f_ and *E*_a_ for a film with ZrO_2_ would be higher compared to those measured on a PbZrO_3_ film with fewer inclusions. The effects of the presence of a layer of ZrO_2_ on *E*_f_ and *E*_a_ are calculated in the Supporting Information and can result in tens to hundred kV/cm variations, corresponding to ∼50% overestimation of the transition fields in this case. Practically speaking, removal of the zirconia layer is expected to result in ∼30% decreases in the measured *E*_f_ and *E*_a_. These results are consistent with the decreases in *E*_f_ and *E*_a_ observed after the removal of surface nanocrystals by PbO capping: by 8% and 11%, respectively, for the nonseeded films (Figures 2b and 4b) and both by 9% for the PbO-seeded films (Figure 6). These values are lower than the decrease predicted using the in series capacitor model, possibly due to the more complex distribution of zirconia throughout the films, as observed by SEM, compared to the simplistic representation of ZrO_2_ as a continuous layer in Figure S9a. Indeed, the surface nanocrystals on the as-crystallized film do not constitute a uniform layer and are predominantly located at the grain boundaries (Figure 3a). However, nanocrystals are also observed at buried crystallization interfaces (Figure 2c). A more realistic model would consider a discontinuous layer of ZrO_2_ nanocrystals and multiple ZrO_2_ capacitors arranged both in series and in parallel with PbZrO_3_. Hence, while the ≈50% variation is an overestimation of the effect of the presence of zirconia on *E*_f_ (and *E*_a_) by our model, we argue that ignoring the presence of nanocrystals in the films results in a systematic and incorrect evaluation of the these critical field for phase transition. The observed changes in *E*_f_ and *E*_a_ correlated with the absence or presence of zirconia nanocrystals, not otherwise discoverable in XRD, highlights once again the need for our community to specifically evaluate the microstructure of PbZrO_3_ thin films for such secondary phases.

## Summary and Conclusions

We reported here on Pb loss and compensation strategies to address the associated challenges with the microstructure and ultimately the functional response of chemical solution processed PbZrO_3_ thin films. These strategies included (1) Pb overstoichiometry in the precursor solution; (2) PbO “capping” layer after the final PbZrO_3_ crystallization; (3) PbO layer insertion after each PbZrO_3_ crystallization; and (4) PbO “seed” layer at the interface with the bottom electrode, before deposition of the first PbZrO_3_ film. Although X-ray diffraction was unable to detect and/or identify any nonperovskite phases, nanocrystals with bright SEM contrast, distinct from the bulk PbZrO_3_, were observed on the films’ surface or at specific depths within the films’ cross-section. These nanocrystals were identified as pure ZrO_2_ through an HR-STEM analysis. HAADF–STEM EDS maps highlighted an overall Pb deficiency at, or in proximity of, each crystallization interface. However, Pb-rich regions were also observed in proximity of the Pb-deficient/Zr-rich interfaces, suggesting that Pb compensation through the precursor solution Pb overstoichiometry was insufficient to fully compensate for Pb-loss during crystallization. Therefore, subsequent PbZrO_3_ deposited layers could provide only a limited amount of Pb (through the overstoichiometric solutions) resulting only in partial transformation of ZrO_*x*_ into perovskite PbZrO_3_. Introducing an additional PbO layer, deposited and crystallized after each PbZrO_3_ crystallization, was effective for substantially reducing the presence of ZrO_*x*_ nanocrystals. Nonetheless, this Pb compensation approach resulted in inclusion of {001}_o_-oriented perovskite grains and, hence, is a suitable strategy only for processing PbZrO_3_ films that are highly {001}_o_-oriented (which can be further enhanced through the use of a PbO seed layer before PbZrO_3_ deposition) or randomly oriented lead zirconate films. Highly 042_o_-oriented films can be processed via chemical solution deposition, with appropriate Pb overstoichiometry of the precursor solution, on high quality platinized Si substrates. However, such films will either show ZrO_*x*_ nanocrystals in proximity of crystallization interfaces or see reduced crystallographic orientation through PbO introduction after each PbZrO_3_ crystallization. Presence of ZrO_*x*_ nanocrystals is accompanied by an increase in the measured antipolar-to-polar and polar-to-antipolar transition field values and a decrease of the saturation polarization. Therefore, we strongly advocate for any work reporting functional properties of perovskite PbZrO_3_ films to include microstructural evaluation of the films to either rule out the presence of secondary phases or enable the community to contextualize the reported transition fields.

## Methods

### PbZrO_3_ Thin Film Processing

0.25 M PbZrO_3_ precursor solutions with 35 mol %, 37.5 mol %, or 40 mol % Pb excess were prepared in-house, using a 2-methoxyethanol (2MOE)-based chemical solution processing route reported previously.^22^ Lead­(II) acetate trihydrate and zirconium­(IV) propoxide were dissolved in 2MOE in separate flasks. The Pb precursor was stirred for 15 min then vacuum distilled at 120 °C until the flask content was dehydrated (indicated by the onset of transformation into a white foam), while the Zr precursor was stirred at 115 °C for 20 min. The Zr precursor was subsequently added to the flask containing the Pb precursor, and the solution was mixed at 115 °C for 4 h. Finally, the solution was diluted to the desired concentration with 2MOE. For deposition of seed and capping layers, PbO precursor solutions with concentrations of 0.08 and 0.14 M were prepared by dehydrating the Pb precursor and redissolving it in 2MOE.

PbZrO_3_ films were deposited on Pt/Ti/SiO_2_/Si substrates. The substrates were platinized in-house, and rocking curves measured for the Pt(111)_pc_ peak had full width at half-maximum values ≤4. The PbZrO_3_ solution was filtered through a 0.10 μm filter and deposited by spin-coating at 3000 rpm for 30 s onto the substrate. Each deposited film was dried at 150 °C for 1 min and subsequently pyrolyzed at 400 °C for 1 min, with each of these two heat treatment steps performed on a separate hot plate. After every two layers (each dried and pyrolyzed after deposition), the films were crystallized by rapid thermal annealing at 725 °C for 70 s. The process was repeated three times until six layers of PbZrO_3_ had been deposited (crystallized three times) to form ∼300 nm thick PbZrO_3_ films. PbO layers were spun-coated, dried, and pyrolyzed under the same conditions as the PbZrO_3_ layers and crystallized at 700 °C for 70 s. The PbO precursor solutions used for seed layers were 0.14 M, and those used for intermediate and capping layers were all 0.08 M. After the crystallization of each PbO capping layer, the surface of the film was washed multiple times with glacial acetic acid in order to remove eventual residual PbO.

### X-ray Diffraction

Crystallographic phase identification and preferred orientation were performed using a PANalytical Empyrean X-ray diffractometer for thin film scans and a Rigaku SE X-ray diffractometer for grazing incidence scans. Monochromated incident Cu Kα X-rays (λ = 1.540598) were used at 45 kV and 40 mA. Thin film 2θ scans were performed from 20° to 60° with a step size of 0.01° and a scan rate of 18.87 s/step. A 20 mm mask and 1° antiscatter slit were used for the incident beam, and a 0.04 rad Soller slit, 1° antiscatter slit, and 7.5 mm divergence slit were used for the diffracted beam. The degree of crystallographic orientation for specific families of directions was characterized using the Lotgering factor (LF).^38^ The powder diffraction file (PDF) 01-089-1296 obtained from the PDF database was used as a reference for peak intensities of randomly oriented PbZrO_3_.

### Electron Microscopy

Film cross sections and surfaces were imaged using a Hitachi SU8230 field-emission scanning electron microscope (SEM). For all micrographs, 5 kV accelerating voltage, 15 μA current, and both the upper and lower secondary electron detectors were used. Film thicknesses were evaluated from cross-sectional SEM images. Samples for transmission electron microscopy (TEM) were prepared using a Tescan focused ion beam (FIB)-secondary electron microscope Lyra 3. High-angle annular dark-field scanning transmission electron microscopy (HAADF–STEM) was performed on a Thermo Fisher Scientific Talos F200X instrument for imaging and EDS of film cross sections. Further fine polishing of lamella was performed with Ar plasma in a Fishione Nanomill; the relative thickness was assessed using *t*/λ mapping in the energy filtered TEM mode at the FEI Titan TEM at 300 kV. High-resolution HAADF–STEM imaging was performed with the use of the NION UltraSTEM operated at 200 kV, ∼25 pA. Parameters for the ab initio image simulations were selected to be close to the experimental ones—a convergence angle of 30 mrad, a collection angle of 99–200 mrad, a thickness of 10 nm, and 20 frozen phonons in use. abTEM software^55^ was used for these simulations with the ase^56^ library for the automated atomic structures generation and VESTA 3 ^57^ for the structure drawings.

### Dielectric Characterization

Buffered oxide etchant (6:1) was used to expose the bottom Pt electrode. Parallel plate capacitor structures were formed by the sputter deposition of ∼200 μm wide circular top Pt electrodes using a metallic shadow mask. The samples were annealed at 400 °C for 10 min after top Pt deposition to improve the metal layer’s contact with the film. A Radiant Precision Multiferroic II tester was used for characterization of the ferroelectric properties. Using a double bipolar sine wave applied at 4 kHz, polarization–electric field (*P*–*E*) hysteresis loops were measured and the corresponding switching current–electric field loops (*I*–*E*) were obtained. Measurements were performed at up to 12 V for 042_o_-oriented PbZrO_3_ and up to 20 V for {001}_o_-oriented PbZrO_3_. Due to film thickness variations, these applied voltages correspond to ∼360 kV/cm ± 15 kV/cm and ∼440 kV/cm ± 30 kV/cm, respectively. Antipolar-to-polar and polar-to-antipolar transition electric field values, *E*_f_ and *E*_a_, respectively, were extracted from the fitted switching current peaks in the *I*–*E* loops. Saturation polarization values were taken as the intercept of the tangent to the return curve at saturation at zero field.

## Supplementary Material



## Notes

Switching current-electric field responses of all films; grazing incident X-ray diffraction (GIXRD) pattern of the as-crystallized PbZrO_3_ processed with 40% Pb excess; scanning electron microscope (SEM) surface image and XRD pattern of the bare Pt bottom electrode; SEM surface image, GIXRD pattern, and energy dispersive X-ray spectroscopy (EDS) maps of the 0.14 M PbO seed layer; high-resolution high-angle annular dark-field scanning transmission electron microscope (HAADF–STEM) image of a crystallization interface in PbZrO_3_ processed with a 0.14 M PbO seed and 40% Pb excess; and HAADF–STEM EDS and high-resolution STEM images of Pb-rich and Pb-deficient inclusions in PbZrO_3_ processed with a 0.14 M PbO seed and 40% Pb excess (PDF)

## References

[1] Randall C. A., Fan Z., Reaney I., Chen L.-Q., Trolier-McKinstry S. (2021). Antiferroelectrics
History, Fundamentals, Crystal Chemistry, Crystal Structures, Size
Effects, and Applications. J. Am. Ceram. Soc..

[2] Reaney I. M., Taylor D. V., Brooks K. G. (1998). Ferroelectric PZT Thin Films by Sol-Gel
Deposition. J. Sol-Gel Sci. Technol..

[3] Lefevre M. J., Speck J. S., Schwartz R. W., Dimos D., Lockwood S. J. (1996). Microstructural
Development in Sol-Gel Derived Lead Zirconate Titanate Thin Films:
The Role of Precursor Stoichiometry and Processing Environment. J. Mater. Res..

[4] Chen S.-Y., Chen I. W. (1994). Temperature–Time
Texture Transition of Pb­(Zr_1‑x_Ti_x_)­O_3_ Thin Films: I, Role
of Pb-Rich Intermediate Phases. J. Am. Ceram.
Soc..

[5] Chen S.-Y., Chen I. W. (1994). Temperature–Time
Texture Transition of Pb­(Zr_1–x_Ti_x_)­O_3_ Thin Films: II, Heat
Treatment and Compositional Effects. J. Am.
Ceram. Soc..

[6] Brennecka G. L., Ihlefeld J. F., Maria J.-P., Tuttle B. A., Clem P. G. (2010). Processing
Technologies for High-Permittivity Thin Films in Capacitor Applications. J. Am. Ceram. Soc..

[7] Liu H., Dkhil B. (2011). A brief review
on the model antiferroelectric PbZrO3 perovskite-like
material. Z. Kristallogr. Cryst. Mater..

[8] Shirane G. (1952). Ferroelectricity
and Antiferroelectricity in Ceramic PbZrO_3_ Containing Ba
or Sr. Phys. Rev..

[9] Si Y., Zhang T., Liu C., Das S., Xu B., Burkovsky R. G., Wei X.-K., Chen Z. (2024). Antiferroelectric oxide
thin-films: Fundamentals, properties, and applications. Prog. Mater. Sci..

[10] Hao X., Zhai J., Kong L. B., Xu Z. (2014). A Comprehensive Review
on the Progress of Lead Zirconate-Based Antiferroelectric Materials. Prog. Mater. Sci..

[11] Schneller, T. ; Waser, R. ; Kosec, M. ; Payne, D. Chemical Solution Deposition of Functional Oxide Thin Films; Springer, 2013; pp 1–796.

[12] Polli A. D., Lange F. F., Levi C. G. (2000). Metastability
of the Fluorite, Pyrochlore,
and Perovskite Structures in the PbO—ZrO_2_—TiO_2_ System. J. Am. Ceram. Soc..

[13] Polli A. D., Lange F. F. (1995). Pyrolysis of Pb­(Zr_0.5_Ti_0.5_)­O_3_ Precursors: Avoiding Lead
Partitioning. J. Am. Ceram. Soc..

[14] Fox G. R., Krupanidhi S. B., More K. L., Allard L. F. (1992). Composition/Structure/Property
Relations of Multi-Ion-Beam Reactive Sputtered Lead Lanthanum Titanate
Thin Films: Part I. Composition and Structure Analysis. J. Mater. Res..

[15] Kwok C. K., Desu S. B. (1991). Role of Oxygen Vacancies on the Ferroelectric Properties
of PZT Thin Films. MRS Online Proc. Libr..

[16] Reaney I. M., Brooks K., Klissurska R., Pawlaczyk C., Setter N. (1994). Use of Transmission Electron Microscopy for the Characterization
of Rapid Thermally Annealed, Solution-Gel, Lead Zirconate Titanate
Films. J. Am. Ceram. Soc..

[17] Tani T., Payne D. A. (1994). Lead Oxide Coatings
on Sol–Gel-Derived Lead
Lanthanum Zirconium Titanate Thin Layers for Enhanced Crystallization
into the Perovskite Structure. J. Am. Ceram.
Soc..

[18] Xu B., Cross L. E., Ravichandran D. (1999). Synthesis
of Lead Zirconate Titanate
Stannate Antiferroelectric Thick Films by Sol-Gel Processing. J. Am. Ceram. Soc..

[19] Snow G. S. (1973). Fabrication
of Transparent Electrooptic PLZT Ceramics by Atmosphere Sintering. J. Am. Ceram. Soc..

[20] Härdtl K. H., Rau H. (1969). PbO Vapour Pressure
in the Pb­(Ti_1–x_)­O_3_ System. Solid State Commun..

[21] Ikeda T., Okano T., Watanabe M. (1962). A Ternary
System PbO–TiO_2_–ZrO_2_. Jpn. J. Appl.
Phys..

[22] Yao Y., Gallego M., Bassiri-Gharb N. (2021). Effects of Nb and Mg Doping on the
Dielectric and Electromechanical Properties of PbZrO_3_ Thin
Films. J. Eur. Ceram. Soc..

[23] Gates-Rector S., Blanton T. (2019). The Powder Diffraction
File: A Quality Materials Characterization
Database. Powder Diffr..

[24] Trolier-McKinstry S., Muralt P. (2004). Thin Film Piezoelectrics for MEMS. J. Electroceram..

[25] Zhang C., Zhang X., Zhang B., Yin C., Zhang Y., Zhang Y., Zhang T., Cui Y., Chi Q. (2024). High-Energy
Storage Performance Achieved in PbZrO_3_ Thin Films via Li^+^ Doping and Low-Temperature Annealing. Thin Solid Films.

[26] Yao Y., Naden A., Tian M., Lisenkov S., Beller Z., Kumar A., Kacher J., Ponomareva I., Bassiri-Gharb N. (2023). Ferrielectricity in the Archetypal
Antiferroelectric,
PbZrO_3_. Adv. Mater..

[27] Zhang T. F., Tang X. G., Liu Q. X., Jiang Y. P., Jiang L. L., Luo L. (2016). Optical and Dielectric Properties of PbZrO_3_ Thin Films
Prepared by a Sol–Gel Process for Energy-Storage Application. Mater. Des..

[28] Alkoy E. M., Shiosaki T. (2008). Electrical Properties
and Leakage Current Behavior
of Un-Doped and Ti-doped Lead Zirconate Thin Films Synthesized by
Sol–Gel Method. Thin Solid Films.

[29] Alkoy E., Alkoy S., Shiosaki T. (2005). Microstructure
and Crystallographic
Orientation Dependence of Electrical Properties in Lead Zirconate
Thin Films Prepared by Sol-Gel Process. Jpn.
J. Appl. Phys..

[30] Muralt P., Maeder T., Sagalowicz L., Hiboux S., Scalese S., Naumovic D., Agostino R. G., Xanthopoulos N., Mathieu H. J., Patthey L., Bullock E. L. (1998). Texture
Control
of PbTiO_3_ and Pb­(Zr,Ti)­O_3_ Thin Films with TiO_2_ Seeding. J. Appl. Phys..

[31] Swartz S., Shrout T. R. (1982). Fabrication of Perovskite Lead Magnesium Niobate. Mater. Res. Bull..

[32] Gueye I., Le Rhun G., Gergaud P., Renault O., Defay E., Barrett N. (2016). Chemistry of Surface Nanostructures in Lead Precursor-Rich
PbZr_0.52_Ti_0.48_O_3_ Sol–Gel Films. Appl. Surf. Sci..

[33] Wang Z. j., Maeda R., Kikuchi K. M. (2000). Electron
Microscopic Observation
of the Microstructure of Sol-Gel Derived PZT Thin Film. J. Jpn. Inst. Metals.

[34] Lima E. C., Araujo E. B., Eiras J. A. (2020). On the
Influence of Pyrochlore Phase
on Ferroelectric and Dielectric Properties of PZT Thin Films. Ferroelectrics.

[35] Brennecka G. L., Tuttle B. A. (2007). Fabrication of Ultrathin
Film Capacitors by Chemical
Solution Deposition. J. Mater. Res..

[36] Brennecka G., Parish C., Tuttle B., Brewer L., Rodriguez M. (2008). Reversibility
of the Perovskite-to-Fluorite Phase Transformation in Lead-Based Thin
and Ultrathin Films. Adv. Mater..

[37] Parish C. M., Brennecka G. L., Tuttle B. A., Brewer L. N. (2008). Quantitative
Chemical
Analysis of Fluorite-to-Perovskite Transformations in (Pb,La)­(Zr,Ti)­O_3_ PLZT Thin Films. J. Mater. Res..

[38] Lotgering F. K. (1959). Topotactical
Reactions with Ferrimagnetic Oxides Having Hexagonal Crystal Structures—I. J. Inorg. Nucl. Chem..

[39] Chen S., Chen I. (1998). Texture Development,
Microstructure Evolution, and Crystallization
of Chemically Derived PZT Thin Films. J. Am.
Ceram. Soc..

[40] Gong W., Li J., Chu X., Gui Z., Li L. (2004). Preparation and Characterization
of Sol-Gel Derived (100)-Textured Pb­(Zr,Ti)­O_3_ Thin Films:
PbO Seeding Role in the Formation of Preferential Orientation. Acta Mater..

[41] Bastani Y., Schmitz-Kempen T., Roelofs A., Bassiri-Gharb N. (2011). Critical Thickness
for Extrinsic Contributions to the Dielectric and Piezoelectric Response
in Lead Zirconate Titanate Ultrathin Films. J. Appl. Phys..

[42] Nguyen M. D., Trinh T. T., Dang H. T., Vu H. N. (2020). Understanding the
Effects of Electric-Field-Induced Phase Transition and Polarization
Loop Behavior on the Energy Storage Performance of Antiferroelectric
PbZrO_3_ Thin Films. Thin Solid Films.

[43] Lisenkov S., Yao Y., Bassiri-Gharb N., Ponomareva I. (2020). Prediction
of High-Strain Polar Phases in Antiferroelectric PbZrO_3_ from a Multiscale Approach. Phys. Rev. B.

[44] Aramberri H., Cazorla C., Stengel M., Íñiguez J. (2021). On the Possibility
that PbZrO_3_ Not be Antiferroelectric. npj Comput. Mater..

[45] Milesi-Brault C., Godard N., Girod S., Fleming Y., El Adib B., Valle N., Glinšek S., Defay E., Guennou M. (2021). Critical Field
Anisotropy in the Antiferroelectric Switching of PbZrO_3_ Films. Appl. Phys. Lett..

[46] Tolédano P., Guennou M. (2016). Theory of
Antiferroelectric Phase Transitions. Phys. Rev.
B.

[47] Dewhurst J. K., Lowther J. E. (1998). Relative Stability, Structure, and
Elastic Properties
of Several Phases of Pure Zirconia. Phys. Rev.
B:Condens. Matter Mater. Phys..

[48] Ryuji S., Horiuchi H., Kume S. (1987). ZrO treated
at 600 °C and 6
GPa, HfO_2_ Crystal Structure Refinement. J. Ceram..

[49] Cancarevic, M. ; Zinkevich, M. ; Aldinger, F. ; Materials Science International Team . Phase diagram of the PbO-ZrO2 system: Datasheet from MSI Eureka in SpringerMaterials. https://materials.springer.com/msi/phase-diagram/docs/sm_msi_r_10_011410_01_full_LnkDia2 (accessed Dec 01, 2025).

[50] Coulibaly M. D., Borderon C., Renoud R., Gundel H. W. (2020). Enhancement of PbZrO_3_ Polarization
Using a Ti Seed Layer for Energy Storage Application. Thin Solid Films.

[51] Coulibaly M. D., Borderon C., Renoud R., Gundel H. W. (2022). Crystallographic
Orientation Dependence of Ferroelectric Domain Walls in Antiferroelectric
Lead Zirconate Thin Films. Curr. Appl. Phys..

[52] Si Y. (2022). Phase Competition in
High-Quality Epitaxial Antiferroelectric PbZrO_3_ Thin Films. ACS Appl. Mater. Interfaces.

[53] Yu Z., Cai H., Fu Z., Zhang L., Chen X., Wang G., Dong X., Xu F. (2020). Microstructural Evolution in Chemical
Solution Deposited PbZrO_3_ Thin Films of Varying Thickness. J. Appl. Phys..

[54] Wen Z. Y., Ma Y. H., Huang H., Zhao C., Liang R., Liu S., Wang Z. J. (2025). Energy
storage Performance of PbZrO_3_–ZrO_2_ Nanocomposite
Films Prepared by Sol-Gel Method. Mater. Today
Commun..

[55] Madsen J., Susi T. (2021). The abTEM code: Transmission
Electron Microscopy from First Principles. Open
Res. Eur..

[56] Larsen A. H. (2017). The Atomic Simulation
Environment—a Python Library for Working
with Atoms. J. Phys.: Condens. Matter.

[57] Momma K., Izumi F. (2011). VESTA 3 for Three-Dimensional Visualization
of Crystal, Volumetric
and Morphology Data. J. Appl. Crystallogr..

