# Supplementary material for: Chemical Compensation Challenges in Processing Antiferroelectric PbZrO3 Thin Films
Source: ACS Omega. 2025 Jul 5;10(27):29878–87. doi: 10.1021/acsomega.5c04818 (PMC12268465; doi:10.1021/acsomega.5c04818)
Supplement: Supplementary file 1 [file ao5c04818_si_001.pdf]

Supplementary Information
Chemical compensation challenges in processing antiferroelectric $PbZrO_3$ thin films

Milan H. Haddad,[†] Vasily Lebedev,[‡,¶] Kristina Holsgrove,[§] Sergio Rivera-Cruz,[‖] Sarah Stock,[‡,¶] Nikhilesh Maity,[⊥] Sergey Lisenkov,[⊥] Inna Ponomareva,[⊥] Amit Kumar,[§] Lewys Jones,[‡,¶] and Nazanin Bassiri-Gharb[*,†,#]

†*School of Materials Science and Engineering, Georgia Institute of Technology, Atlanta, GA 30332, USA*

‡*School of Physics, Trinity College Dublin, College Green, Dublin 2, Ireland*

¶*Advanced Microscopy Laboratory, Centre for Research on Adaptive Nanostructures & Nanodevices (CRANN), Trinity College Dublin, Dublin 2, Ireland*

§*Centre for Quantum Materials and Technologies, School of Mathematics and Physics, Queen's University Belfast, Main Physics Building, University Road, Belfast, BT7 1NN, Northern Ireland, UK*

‖*Electrical and Computer Engineering Department, University of Puerto Rico-Mayaguez, Mayaguez, 00680, Puerto Rico*

⊥*Department of Physics, University of South Florida, Tampa, FL 33620, USA*

#*G.W. Woodruff School of Mechanical Engineering, Georgia Institute of Technology, Atlanta, GA 30332-0405, USA*

E-mail: nazanin.bassirigharb@me.gatech.edu

Summary of structural and functional properties of $PbZrO_3$ films

Table S1: Summary of $PbZrO_3$ film thicknesses, Lotgering factors, LF_{042} or LF_{001}, transition electric fields, E_f and E_a, and saturation polarization values, P_s.

Bulk Pb excess (mol%)	0.08 M PbO layer(s)	Thickness (nm)	LF_{042}	LF_{001}	E_f (kV/cm)	E_a (kV/cm)	P_s (μC/cm^2)
40%	None	280±5	100%	-	350±7	210±4	45±1
40%	Cap	290±5	100%	-	320±5	180±2	50±3
35%	Intermediate and cap	270±5	85%	-	260±4	140±3	42±1
40%	Seed	300±5	-	92%	570±8	380±3	26±1
40%	Seed and cap	310±5	-	97%	520±10	330±4	25±2

Additional functional and structural characterization of films

Switching current-electric field, *I-E*, loops were used to determine the antipolar-to-polar and polar-to-antipolar transition electric fields, E_f and E_a, respectively, as the peaks of *I-E* loops indicate polarization switching of domains. The *I-E* loops are measured under the same conditions as the polarization-electric field hysteresis, *P-E*, loops, and the data shown in Figure S1a, b, and c correspond to the data shown in Figures 2b and 4b, Figure 6b, and Figure 5b, respectively. Circular Pt electrodes with areas ranging from 0.0003 cm^2 to 0.0005 cm^2 were used for these measurements, hence the variation of the switching current peak areas.

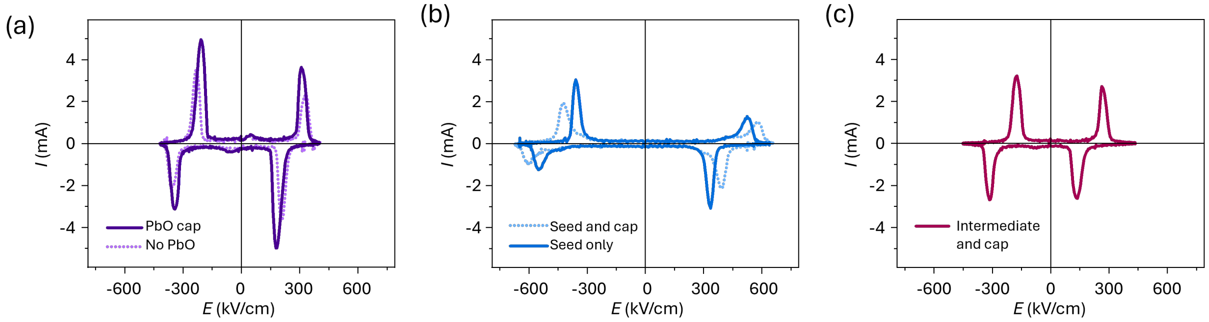

Figure S1: Switching current-electric field, *I-E*, responses of $PbZrO_3$ films processed using (a) 40% Pb excess, both as-crystallized and with a 0.08 M PbO cap, (b) a 0.14 M PbO seed and 40% Pb excess, both as-crystallized and with a 0.08 M PbO cap, and (c) 35% Pb excess and 0.08 M PbO layers after each $PbZrO_3$ crystallization step.

The thin film XRD and grazing-incident XRD (GIXRD) patterns of the $PbZrO_3$ processed with 40% Pb excess are shown in Figure S2. Grazing-incident 2θ scans were performed from 20° to 70° using incidence angle of ω=0.3° with a step size of 0.0048° and a scan rate of 0.17°/second. A 10 mm mask was used for the incident beam, and a 0.04 rad Soller slit was used for the diffracted beam in these measurements. One peak in the GIXRD pattern at 2θ=57.4 ° was not assigned to perovskite $PbZrO_3$ or the substrate.

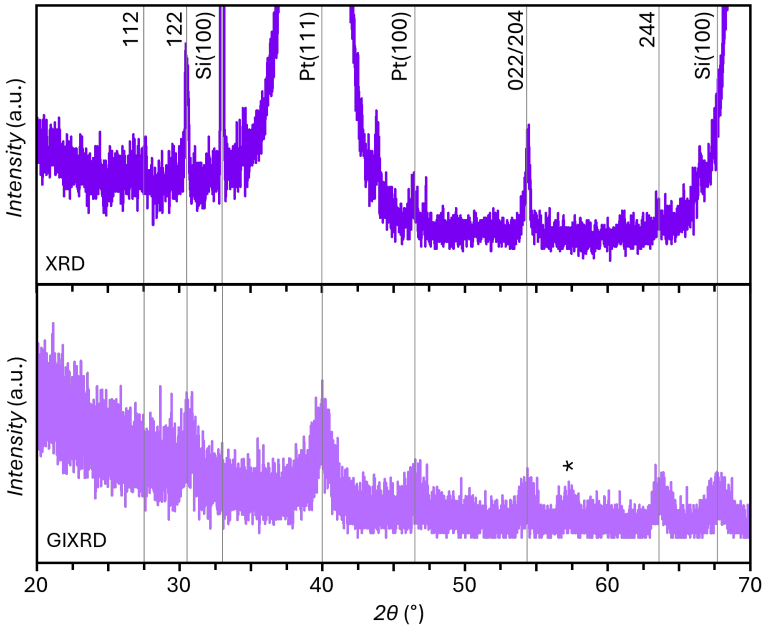

Figure S2: XRD and grazing-incidence XRD (GIXRD) patterns for $PbZrO_3$ processed with 40% Pb excess. The peak labeled with an asterisk (*) is not assigned to perovskite $PbZrO_3$ or the substrate.

Pt bottom electrode and PbO seed layer

Substantial PbO volatilization and Pb diffusion into the substrate can occur during crystallization of the PbO seed, and therefore, the PbO precursor solution concentration was increased from 0.08 M to 0.14 M. The as-deposited Pt bottom electrode has fine grains < 30 nm wide (Figure S3a), whereas the crystallized PbO seed appears as a continuous layer of larger grains (on average more than ∼50 nm wide) with some smaller faceted crystallites possibly protruding on the surface (Figure S3b). The XRD pattern of the as-deposited Pt bottom electrode is shown in Figure S3c. For insight into the crystallographic orientation of the deposited PbO layer, GIXRD was performed after the seed layer crystallization. However, only one peak is observed at 2θ=67.9° (Figure S3d). This peak could not be assigned to the perovskite $PbZrO_3$ phase or the substrate and is tentatively assigned as a reflection of the 004 reflection of PbO_2, which has an XRD signal at 2θ=68.2°.[1] Alternatively, this peak may be a reflection of the 400 plane of PbO (2θ=68.3°) (PDF 00-005-0570).[2] No Pt_xPb intermetallic phase – frequently reported to form in PZT[3–5] and PLZT[6] films deposited on platinized substrates – could be matched to the same. Energy-dispersive X-ray spectroscopy (EDS) maps confirm the presence of Pb (as well as Pt and Ti, Figure S3e).

Continuity of crystal lattice

Despite the presence of a Pb-deficient phase at crystallization interfaces, high-resolution HAADF-STEM images indicate continuity of the $PbZrO_3$ lattice across the interfaces: the film on both sides possesses the same $\{001\}_o$ orientation (Figure S4). This result suggests that the Pb-deficient phase does not disrupt templating of subsequent growth of the perovskite phase from the underlying $PbZrO_3$ grain during crystallization of the subsequent $PbZrO_3$ layer. This crystalline continuity is consistent with the high degree of crystallographic orientation observed for the films (Figure 6a).

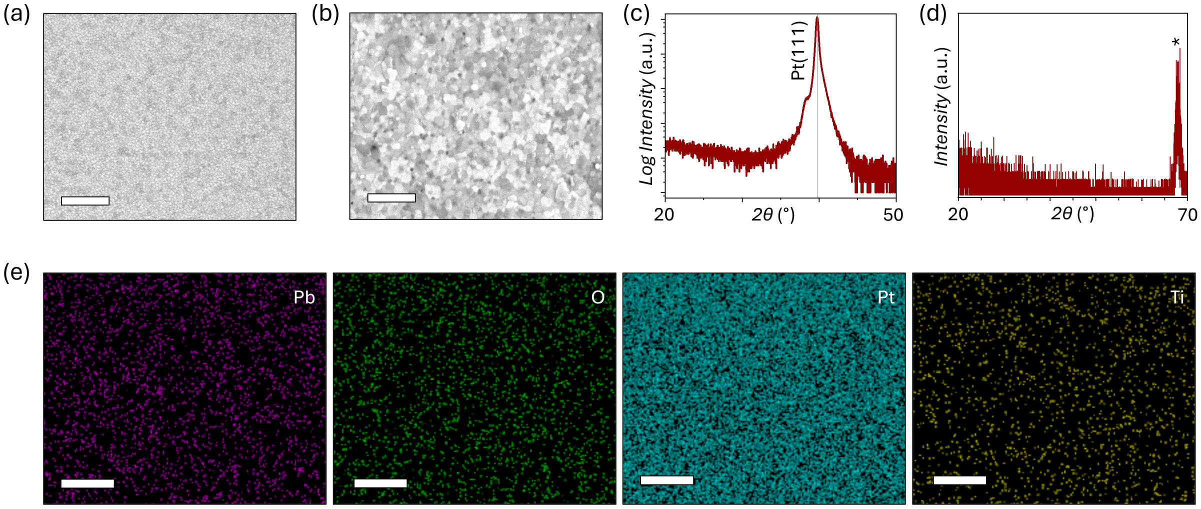

Figure S3: (a,b) SEM surface images, (c) XRD, and (d) grazing incident X-ray diffraction (GIXRD) of (a,c) an as-deposited Pt bottom electrode and (b,d) after deposition and crystallization of a 0.14 M PbO seed layer. (e) The EDS maps of the area shown in (b). The peak labeled with an asterisk (*) in (d) is a potential match to the 004 reflection of PbO_2. Scale bars are 400 nm across all panels.

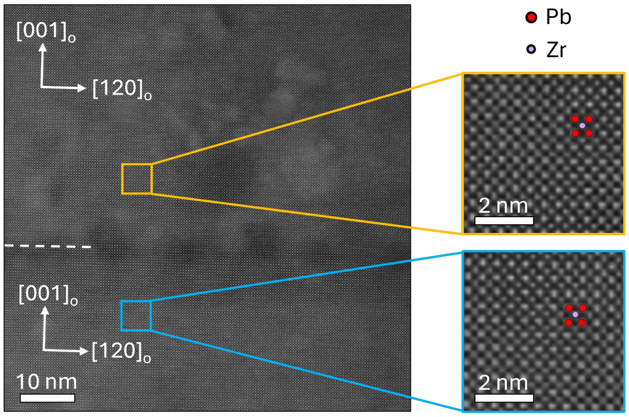

Figure S4: High-resolution HAADF-STEM image across a crystallization interface in $PbZrO_3$ processed with 40% Pb excess and a 0.14 M PbO seed. The crystallization interface is indicated by a white dashed line. Crystallographic directions are given with respect to the orthorhombic system. Scale bar lengths are indicated in the figure.

Pb-rich inclusions

Pb-rich "pockets" were observed in the cross-section of a film processed with a 0.14 M PbO seed and 40% Pb excess (Figure 7c). To confirm the presence of such Pb-rich material, HAADF-STEM analysis was performed on a region directly above a buried crystallization interface. An EDS line scan and mapping show a higher atomic concentration of Pb corresponding to inclusions with bright contrast and a layer with a lower atomic concentration of Pb corresponding to the crystallization interface (Figure S5).

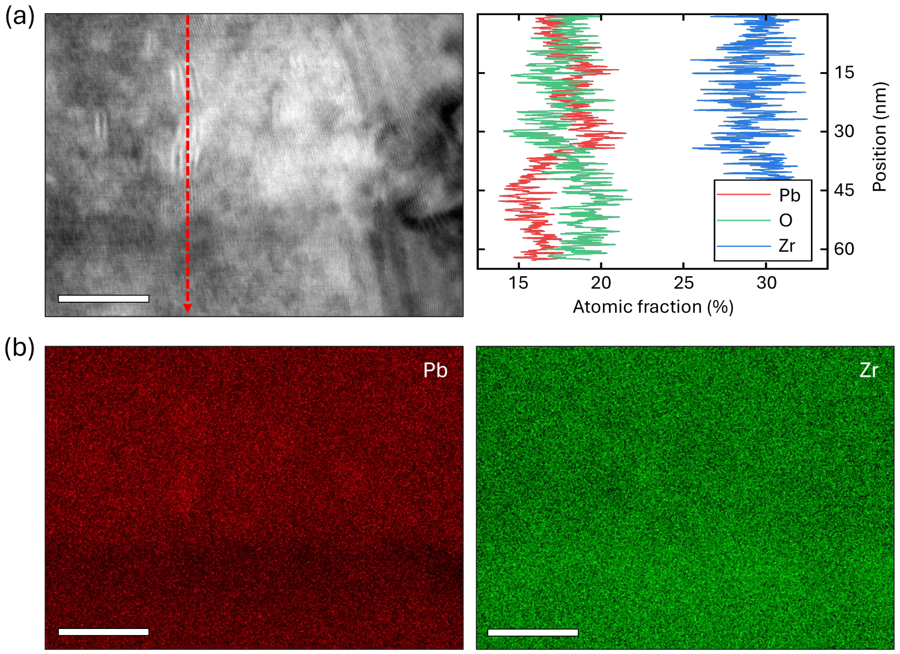

Figure S5: (a) HAADF-STEM image and EDS line profile (marked by a dashed red line) and (b) maps from the film cross-section as shown in (a) of Pb-rich inclusions observed in proximity of a buried crystallization interface in a $PbZrO_3$ film processed with a 0.14 M PbO seed and 40% Pb excess. The X-ray emission lines used were Pt L-α, Ti K-α, Pb L-α, Si K-α, Zr K-α, and O K-α. Scale bars are 50 nm across all panels.

HR-STEM analysis

A series of HR-STEM frames with the dose $\sim 1.3 \cdot 10^5\ e^-/Ang^2$ per frame was acquired from the area of interest shown in Figure S6a. The first 75 frames were distortion corrected and averaged after non-rigid registration using the SmartAlign software from HREM Research.[7] Using the single crystal $PbZrO_3$ matrix area highlighted in Figure S7a as a reference, the relative uncertainty of the STEM image magnification is estimated as $< 2\%$, and the spatial precision as $<$ 7pm (Figure S7b-e). The enlargement from the inclusion (Figure S8a) shows crystallography similar in appearance to $[110]_{fcc}$ with some distortions, specifically the relative shift of atomic columns in the direction labeled as X on Figure S8b.

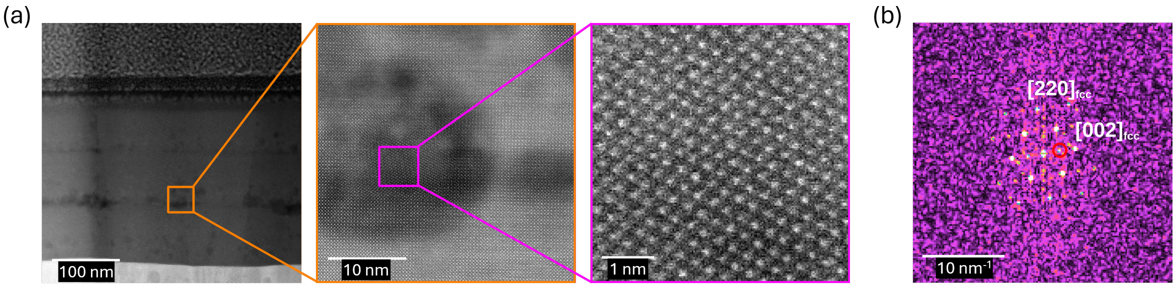

Figure S6: High-resolution STEM of a $PbZrO_3$ film processed with 40% Pb excess and a 0.14 M PbO seed: (a) location of an inclusion within the film, preliminary scan of the inclusion, and the cropped area of the inclusion segment; (b) the corresponding FFT with the preliminary indexing. Scale bar lengths are indicated in each panel.

The lateral size of the inclusion was assessed using the iFFT combined image where different colors correspond to different observed diffraction patterns, and the color code follows the FFT on the inset. An enlargement of the center of the high-pass filtered image (Figure S8b) reveals a 70 ± 30 pm systematic displacement in the X direction (d_a) with an average period of 5 Å(Figure S8c). Meanwhile, the projection on the Y direction (Figure S8d) demonstrates evenly spaced peaks in the intensity line-profiles with a period of 3.6 Å. The presence of such a displacement on the acquired image is confirmed by further analysis of the relative positions of the atomic columns (Figure S7, Figure S9). The observed values of interatomic distances and systematic displacements with respect to the estimated precision

allows us to propose the orthorhombic Ortho-I[8,9] phase of ZrO_2 (space group *Pbcm*) as the best fit; other potential matches are $Pbc2_1$ or *Pbca* of the same structure (Table S2). The proposed structures and corresponding simulated HAADF-STEM images are shown in Figure 8d-g.

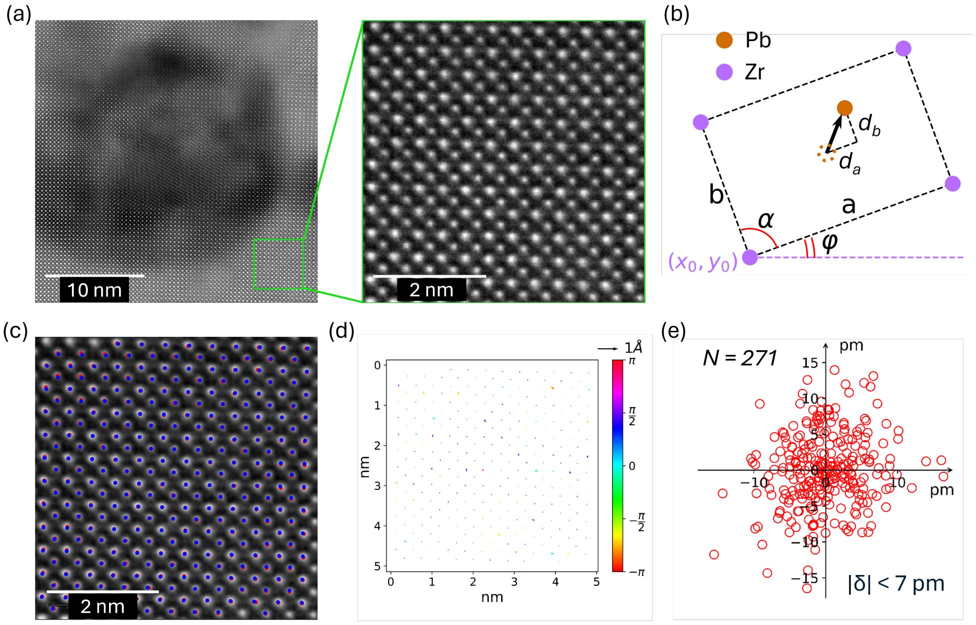

Figure S7: High-resolution STEM of the $PbZrO_3$ matrix area in proximity of the inclusion shown in Figure S6a: (a) multi-frame dataset, averaged with the non-rigid alignment, and reference segment of $PbZrO_3$; (b) model proposed for the atomic column positions fit; (c) the reference segment with overlaid atomic column positions as detected by atomap (red) and as determined by refinement using the model exemplified in (b) (blue); (d) vector map of differences between the model and detected positions; (e) plot of residuals and the average geometric distance between expected and observed positions. Scale bar lengths are indicated in each relevant panel.

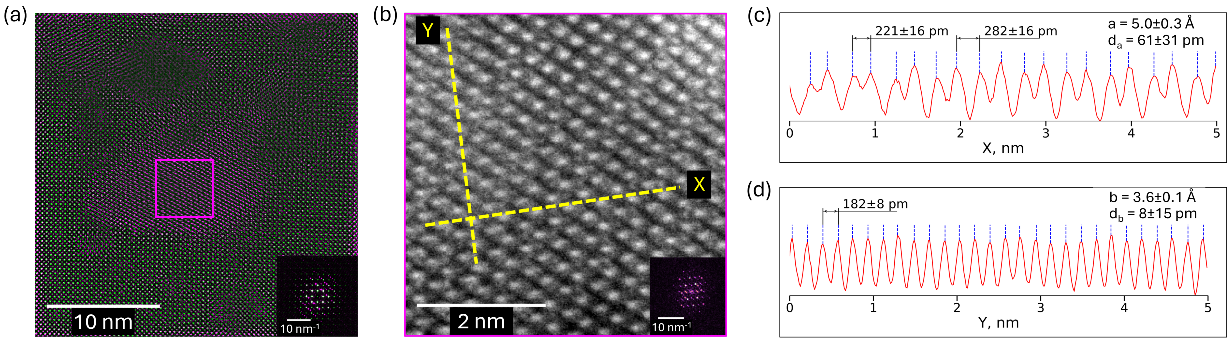

Figure S8: (a) High-resolution STEM preliminary scan of the inclusion shown in Figure S6a overlaid with false coloring indicating different diffraction patterns and corresponding with the FFT (inset), (b) enlargement of the inclusion area with the FFT inset, and projections of the high-pass filtered image segment of the inclusion along the (b) X and (c) Y axes. X and Y directions are indicated in (b). Scale bar lengths are indicated in each relevant panel.

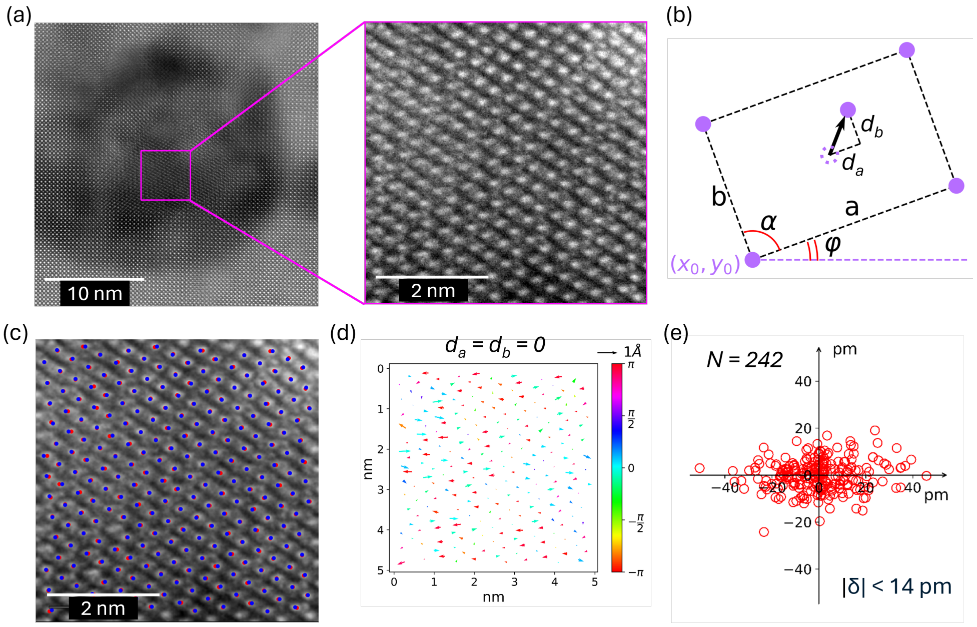

Figure S9: High-resolution STEM of the inclusion shown in Figure S6a: (a) multi-frame dataset, averaged with the non-rigid alignment, and segment of the inclusion (b) model proposed for the atomic column positions fit; (c) the inclusion segment with overlaid atomic column positions as detected by atomap (red) and as determined by refinement using the model exemplified in (b) (blue); (d) vector map of differences between the constrained model and detected positions; (e) plot of residuals and the average geometric distance between expected and observed positions. Scale bar lengths are indicated in each relevant panel.

For phase identification, a preliminary list of potential matches was created with the use

of the free Crystallography Open Database (COD)[10] and constraints arising from the FFT of the inclusion (Figure SS6). The chemical composition was restricted to Pb, Zr, Pt, and O. The following constraints were used to find a suitable *uvw*:

- $1/d_1 = 1.82 \pm 0.1\text{Å}^{-1}$ should be observed;
- $1/d_2 = 2.5 \pm 0.1\text{Å}^{-1}$ should be observed;
- both $h_1k_1l_1$ and $h_2k_2l_2$ are expected to be even to allow centering;
- the angle between $1/d_1$ and $1/d_1$ should be $90° \pm 5°$

This procedure allowed us to select potential crystallographic matches, namely - 'Ortho-I' ZrO_2 and other closely related phases of zirconia, α-PbO, and α-PbO_2. The full list of potential matches was then created based on literature data of similar structures; the ZrO_2 structures notation was based on the work by Dewhurst and Lowther[8] and partially on that of Kersch and Falkowski.[11] The fit outcomes of the simulated structures and orientations detailed in Table S2 allowed us to arrange potential matches by the average difference of a,b values from measured ones, rule out ones with a or b absolute deviation >5%, and select ones with $d_b <$ 7 pm and $d_a >$6 pm. As a result, only three structures, all corresponding to the 'Ortho-I' ZrO_2 phase, remained as potential matches with the observed data.

The detailed data sheet is available; for each of the structures and orientations from the Table S2, the refined parameters a, b, d_a, and d_b (see Figure S9c for details) are provided in the 'Full output' sheet; filtered records after thresholds applied to lattice parameters and modulations are provided in the 'ab filtered' and 'dadb filtered' datasheets, correspondingly.

Table S2: Structures and orientations for which HAADF-STEM images with the limited source size of 0.5 Å were simulated in abTEM and analyzed with the same model as proposed in Figure S7.

Composition	Crystal Structure	Space Group	Orientations	Source (doi)
ZrO_2	Monoclinic	$P2_1/c$	$[101]$, $[10\bar{1}]$	[12]
ZrO_2	Tetragonal	$P4_2/nmc$	$[100]$, $[111]$, $[11\bar{1}]$, $[\bar{1}11]$	[13]
ZrO_2	Cubic	$Fm\bar{3}m$	$[110]$	[14]
ZrO_2	Ortho-I	$Pbca$ (antipolar)	$[012]$, $[01\bar{2}]$, $[210]$, $[2\bar{1}0]$, $[101]$, $[10\bar{1}]$	[11]
ZrO_2	Ortho-I	$Pbca$ (nonpolar)	$[201]$, $[20\bar{1}]$, $[021]$, $[02\bar{1}]$	[11]
ZrO_2	Ortho-I	$Pbcm$	$[011]$, $[01\bar{1}]$, $[101]$, $[10\bar{1}]$, $[110]$, $[1\bar{1}0]$	[9]
ZrO_2	Ortho-I	$Pca2_1/Pbc2_1$	$[011]$, $[01\bar{1}]$, $[101]$, $[10\bar{1}]$, $[110]$, $[1\bar{1}0]$	[15]
ZrO_2	Ortho-II	$Pnam$	$[001]$	[16]
PbO_2	Ortho-III	$Pbcn$	$[110]$, $[1\bar{1}0]$, $[101]$, $[10\bar{1}]$	[1]
PbO_2	Cubic	$Fm\bar{3}m$	$[110]$	[17]
PbO	Tetragonal-II	$P4/nmm$	$[100]$, $[111]$	[18]
PbO	Tetragonal-III	$Pbma$	$[810]$, $[8\bar{1}0]$	[19]

Effect of ZrO_2 nanoparticles on measured phase transition electric fields

We now consider the effects of the presence of ZrO_2 nanocrystals on the functional properties of the films. In first approximation, the surface nanocrystals can be considered as a layer of ZrO_2 at the interface between the metal top electrode and the $PbZrO_3$ film (Figure S10a). The metal-insulator(s)-metal stack is considered in a parallel-plates capacitor approximation. Therefore, ZrO_2 and $PbZrO_3$ can be modeled as two capacitors in series (Figure S10b), where the capacitance of the overall parallel-plate capacitor between the top and bottom electrode, C_{film}, is:

$$\frac{1}{\mathrm{C_{film}}} = \frac{1}{\mathrm{C_{PbZrO_3}}} + \frac{1}{\mathrm{C_{ZrO_2}}} \tag{1}$$

$$\frac{1}{\mathrm{C_{film}}} = \frac{\mathrm{d_{film}}}{\varepsilon_{\mathrm{film}}\varepsilon_0 A} = \frac{\mathrm{d_{PbZrO_3}}}{\varepsilon_{\mathrm{PbZrO_3}}\varepsilon_0 A} + \frac{1}{\mathrm{C_{ZrO_2}}} \tag{2}$$

where C_i, d_i, and ε_i are the capacitance, thickness, and relative dielectric permittivity of each material, and ε_0 is the dielectric permittivity of free space. A is the electrode area for the parallel plate capacitor, and throughout this work it is 0.0005 cm^2.

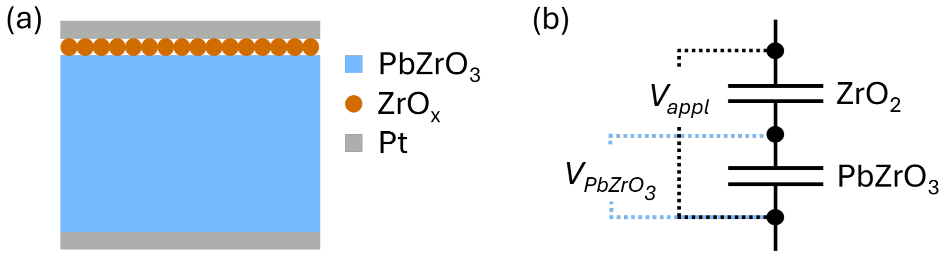

Figure S10: (a) Schematic of a $PbZrO_3$ film with surface ZrO_2 sandwiched between two Pt electrodes as a parallel-plate capacitor and (b) the corresponding diagram where $PbZrO_3$ and ZrO_2 are modeled as two capacitors in series, with the applied voltage, V_{appl}, and the effective voltage across the $PbZrO_3$, V_{PbZrO_3}, indicated.

The presence of a second capacitor in series with lead zirconate can therefore result into a voltage drop with respect to the overall applied voltage. As shown in Figure S10b, the

overall measured film capacitance, C_{film}, the total voltage applied, V_{appl}, and the voltage drops across each of the two in series capacitors, V_{PbZrO_3} and V_{ZrO_x}, are related by:

$$\mathrm{V_{appl} = V_{PbZrO_3} + V_{ZrO_2} = V_{PbZrO_3} + V_{appl}\frac{C_{film}}{C_{ZrO_2}}} \tag{3}$$

Otherwise put, in absence of a zirconia layer, the resulting voltage across the $PbZrO_3$ is simply due to the applied voltage, and $V_{appl} = V_{PbZrO_3}$. However, in the presence of a zirconia layer, the voltage drop across this layer, V_{ZrO_2}, results into a lower effective V_{PbZrO_3}, which can be calculated using Equation 3. In this case, V_{ZrO_2}/V_{PbZrO_3} can be considered as the proportion by which the applied voltage must be increased for the voltage across $PbZrO_3$ in a film containing a zirconia layer to equal that of a film without any secondary phases.

According to Equations 2 and 3, voltage drop across the zirconia layer can be evaluated if d_{film}, d_{PbZrO_3}, ε_{film}, and ε_{PbZrO_3} are known. In this work, the ZrO_2 layer was indistinguishable from the $PbZrO_3$ in the cross sectional SEM images of the as-crystallized film (Figure 2c). Therefore, we assume $d_{film} \approx d_{PbZrO_3}$, and the thickness of the as-crystallized film, 280±5 nm, is considered as the total film thickness. Given that our PbO capped films show a more limited amount of nanocrystals on the surface compared to the equivalent as-crystallized films (Figure 3), we leverage the nonlinear dielectric responses of the two films processed with 40% Pb excess to evaluate ε_{film} and ε_{PbZrO_3} (Figure S11) for the 042-oriented lead zirconate as an example. In these measurements, a 100 nV DC bias was superimposed on a 4 kHz AC voltage, where the latter voltage was increased from 0 V to 14 V in 0.1 V increments.

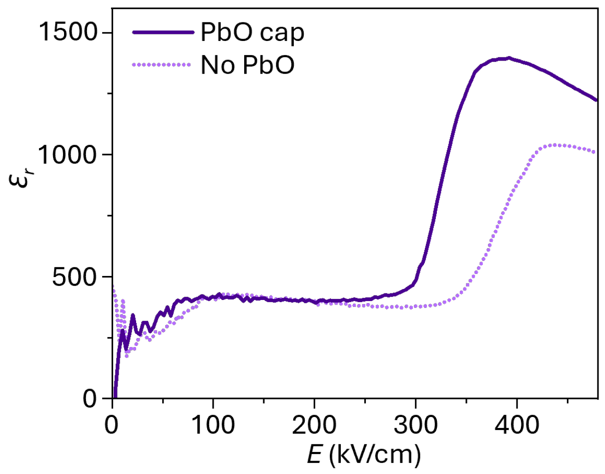

Figure S11: Nonlinear dielectric response as a function of applied electric field for a 285±5 nm thick, 40% Pb excess processed $PbZrO_3$ film. Data are acquired both from the as processed sample and the post-deposition sample recrystallized with a 0.08 M PbO cap. The measurements were performed as a function of 4 kHz AC voltage with a superimposed 100 nV DC voltage.

An initial increase in dielectric permittivity with the applied electric field amplitude is consistent with the observed double switching events (in switching current curves, Figure S1a) in proximity of zero field for 042-oriented films. Such small switching events have been previously assigned to presence of a ferroelectric phase stabilized at low fields: a ferroelectric phase would show signatures of nonlinear and hysteretic motion of domain walls contributing to field-dependent permittivity. At further increase in applied electric field, above approximately 100 kV/cm, the dielectric permittivity plateaus or shows slight decline for both films: such behavior could be associated with a more limited contribution of domain wall motion to the dielectric permittivity at such higher fields. The dielectric permittivity then increases as a function of applied (AC) electric field beyond an inflection point at the onset of the antipolar-to-polar phase transition. At the middle point for each antipolar-to-polar transition, the dielectric permittivity of the sample was evaluated, corresponding to $\varepsilon_{film} = 620 \pm 14$ and $\varepsilon_{PbZrO_3} = 900 \pm 23$, respectively. The reported uncertainty is due to statistical error of the measured functional properties.

We can now attempt to evaluate the effective V_{PbZrO_3} at the measured E_f. V_{appl} is $\approx 9.2\ V$ at the antipolar-to-polar transition of the PbO capped film, i.e., without surface zirconia nanocrystals. In presence of ZrO_2, the voltage drop across this layer is calculated to be $\approx 3.2\ V$, based on Equation 3. Therefore, at an applied $9.2\ V$, the effective voltage on the lead zirconate layer is $V_{PbZrO_3} = 6.0 \pm 0.1\ V$. Otherwise put, use of the applied voltage to evaluate E_f results in a 54% overestimation of the critical field, *IF* there is a continuous zirconia layer in the lead zirconate samples. To confirm the contribution of zirconia to the dielectric response, the dielectric permittivity of ZrO_2, ε_{ZrO_2}, was calculated by considering the thickness of this layer, d_{ZrO_2}, to be the approximated lateral size of the surface nanocrystals, 1–10 nm (Figure 3a). Using ε_{film} and ε_{PbZrO_3} at the high fields corresponding to E_f, ε_{ZrO_2} is evaluated at 7–72 for this thickness range. The nonlinear dielectric responses of these films were also analyzed at low-field ($E = 0$); however, due to high variability of ε_{film} and ε_{PbZrO_3}, the corresponding ε_{ZrO_2} could not be evaluated. The dielectric permittivity of Ortho-I-phased ZrO_2 is reported in literature as ∼30 in the absence of applied field.[20] The range of ε_{ZrO_2} evaluated at high fields here is fairly consistent with this value, considering the extreme possible values of d_{ZrO_2} are used. These results confirm that the presence of ZrO_2 at crystallization interfaces, and reduction of their appearance by Pb compensation, does contribute to the dielectric response of $PbZrO_3$ thin films and substantially affects the effective value of E_f and E_a.

Next, we note that orthorhombic phased ZrO_2 films as thin as 8 nm, similar to the size of the ZrO_2 nanoparticles observed here, have shown ferroelectric polarization switching at room temperature.[21] If the ZrO_2 nanoparticles are ferroelectrically active, then their field-dependent dielectric permittivity[22,23] could further change the effective transition fields observed in the lead zirconate thin films. An increasing dielectric permittivity with applied electric field's amplitude in the zirconia particles would be consistent also with the apparent decrease observed in the dielectric permittivity of the film with no PbO cap. While a quantitative evaluation and separation of such nonlinear dielectric contribution is not within

the scope of the current work, it cannot be ruled out and shall be considered when evaluating literature variations in the reported response of $PbZrO_3$ films.

References

(1) Zaslavskii, A.; Kondrashov, Y. D.; Tolkachev, S. New Modification of Lead Dioxide and the Texture of Anodic Deposits. Dokl. Akad. Nauk. SSSR. pp 559–561.

(2) Gates-Rector, S.; Blanton, T. The Powder Diffraction File: A Quality Materials Characterization Database. *Powder Diffraction* **2019**, *34*, 352–360.

(3) Kaewchinda, D.; Chairaungsri, T.; Naksata, M.; Milne, S.; Brydson, R. TEM Characterization of PZT Films Prepared by a Diol Route on Platinised Silicon Substrates. *Journal of the European Ceramic Society* **2000**, *20*, 1277–1288.

(4) Chen, S.-Y.; Chen, I. W. Temperature–Time Texture Transition of $Pb(Zr_{1-x}Ti_x)O_3$ Thin Films: I, Role of Pb-Rich Intermediate Phases. *Journal of the American Ceramic Society* **1994**, *77*, 2332–2336.

(5) Huang, Z.; Zhang, Q.; Whatmore, R. The Role of an Intermetallic Phase on the Crystallization of Lead Zirconate Titanate in Sol–Gel Process. *Journal of materials science letters* **1998**, *17*, 1157–1159.

(6) Nittala, K.; Brennecka, G. L.; Tuttle, B. A.; Jones, J. L. Phase evolution in Solution Deposited Pb-Deficient PLZT Thin Films. *Journal of Materials Science* **2011**, *46*, 2148–2154.

(7) Jones, L.; Yang, H.; Pennycook, T. J.; Marshall, M. S.; Van Aert, S.; Browning, N. D.; Castell, M. R.; Nellist, P. D. Smart Align — A New Tool for Robust Non-Rigid Registration of Scanning Microscope Data. *Advanced Structural and Chemical Imaging* **2015**, *1*, 1–16.

(8) Dewhurst, J. K.; Lowther, J. E. Relative Stability, Structure, and Elastic Properties of Several Phases of Pure Zirconia. *Physical Review B* **1998**, *57*, 741–747.

(9) Ryuji, S.; Horiuchi, H.; Kume, S. ZrO treated at 600°C and 6GPa, HfO_2 Crystal Structure Refinement. *Journal of the Ceramic Association, Japan* **1987**, *95*, 567–568.

(10) Gražulis, S.; Merkys, A.; Vaitkus, A. In *Handbook of Materials Modeling: Methods: Theory and Modeling*; Andreoni, W., Yip, S., Eds.; Springer International Publishing: Cham, 2020; pp 1863–1881.

(11) Kersch, A.; Falkowski, M. New Low-Energy Crystal Structures in ZrO_2 and HfO_2. *physica status solidi (RRL) – Rapid Research Letters* **2021**, *15*, 2100074.

(12) Smith, D. K.; Newkirk, W. The Crystal Structure of Baddeleyite (Monoclinic ZrO_2) and its Relation to the Polymorphism of ZrO_2. *Acta Crystallographica* **1965**, *18*, 983–991.

(13) Bouvier, P.; Djurado, E.; Ritter, C.; Dianoux, A. J.; Lucazeau, G. Low Temperature Phase Transformation of Nanocrystalline Tetragonal ZrO_2 by Neutron and Raman Scattering Studies. *International Journal of Inorganic Materials* **2001**, *3*, 647–654.

(14) Katz, J. X-Ray Diffraction Powder Pattern of Metastable Cubic ZrO_2. *American Ceramic Society* **1971**, *54*, 531.

(15) Kisi, E. H.; Howard, C. J.; Hill, R. J. Crystal Structure of Orthorhombic Zirconia in Partially Stabilized Zirconia. *Journal of the American Ceramic Society* **1989**, *72*, 1757–1760.

(16) Haines, J.; Léger, J. M.; Hull, S.; Petitet, J. P.; Pereira, A. S.; Perottoni, C. A.; da Jornada, J. A. Characterization of the Cotunnite-Type Phases of Zirconia and Hafnia by Neutron Diffraction and Raman Spectroscopy. *Journal of the American Ceramic Society* **1997**, *80*, 1910–1914.

(17) Liu, L.-G. The High-Pressure Phase Transformations of PbO_2: An In-Situ X-ray Diffraction Study. *Physics and Chemistry of Minerals* **1980**, *6*, 187–196.

(18) Dickinson, R. G.; Friauf, J. B. The Crystal Structure of Tetragonal Lead Monoxide. *Journal of the American Chemical Society* **1924**, *46*, 2457–2463.

(19) Baldinozzi, G.; Raulot, J.-M.; Petricek, V. Reinvestigation of the Incommensurate Structure of PbO. *MRS Online Proceedings Library (OPL)* **2002**, *755*, 1–6.

(20) Zhang, Y.; Chen, H.-X.; Duan, L.; Fan, J.-B.; Ni, L.; Ji, V. A Comparison Study of the Born Effective Charges and Dielectric Properties of the Cubic, Tetragonal, Monoclinic, Ortho-I, Ortho-II and Ortho-III Phases of Zirconia. *Solid State Sciences* **2018**, *81*, 58–65.

(21) Silva, J. P. B.; Istrate, M. C.; Hellenbrand, M.; Jan, A.; Becker, M. T.; Symonowicz, J.; Figueiras, F. G.; Lenzi, V.; Hill, M. O.; Ghica, C.; Romanyuk, K. N.; Gomes, M. J. M.; Martino, G. D.; Marques, L.; MacManus-Driscoll, J. L. Ferroelectricity and Negative Piezoelectric Coefficient in Orthorhombic Phase Pure ZrO_2 Thin Films. *Applied Materials Today* **2023**, *30*, 101708.

(22) Luo, X.; Toprasertpong, K.; Takenaka, M.; Takagi, S. Antiferroelectric Properties of ZrO2 Ultra-Thin Films Prepared by Atomic Layer Deposition. *Applied Physics Letters* **2021**, *118*.

(23) Tasneem, N.; Yousry, Y. M.; Tian, M.; Dopita, M.; Reyes-Lillo, S. E.; Kacher, J.; Bassiri-Gharb, N.; Khan, A. I. A Janovec-Kay-Dunn-Like Behavior at Thickness Scaling in Ultra-Thin Antiferroelectric ZrO2 Films. *Advanced Electronic Materials* **2021**, *7*, 2170049.
